# Outer membrane vesicles secreted by pathogenic and nonpathogenic *Bacteroides fragilis* represent different metabolic activities

**DOI:** 10.1038/s41598-017-05264-6

**Published:** 2017-07-10

**Authors:** Natalya B. Zakharzhevskaya, Anna A. Vanyushkina, Ilya A. Altukhov, Aleksey L. Shavarda, Ivan O. Butenko, Daria V. Rakitina, Anastasia S. Nikitina, Aleksandr I. Manolov, Alina N. Egorova, Eugene E. Kulikov, Innokentii E. Vishnyakov, Gleb Y. Fisunov, Vadim M. Govorun

**Affiliations:** 1 Federal Research and Clinical Centre of Physical-Chemical Medicine Federal Medical Biological Agency, Malaya Pirogovskaya str., 1a, Moscow, 119435 Russian Federation; 2 Moscow Institute of Physics and Technology, Institutskiy Pereulok 9, Dolgoprudny, 141700 Russian Federation; 3 Research Resource Center Molecular and Cell Technologies, Saint-Petersburg State University, Universitetskaya nab. 7-9, Saint-Petersburg, 199034 Russian Federation; 4 Analytical Phytochemistry Laboratory, Komarov Botanical Institute, Prof. Popov Street 2, Saint-Petersburg, 197376 Russia; 5 Shemyakin-Ovchinnikov Institute of Bioorganic Chemistry, Miklukho-Maklaya str. 16/10, Moscow 117997, Russian Federation, Moscow, Russia; 6 Lab of Genome Structural Organization, Institute of Cytology, Russian Academy of Sciences, Saint Petersburg, Russia; 7 Institute of Nanobiotechnologies, Peter the Great St. Petersburg Polytechnic University, Saint Petersburg, Russia; 8 Microbial viruses laboratory, Research Center of Biotechnology RAS, Moscow, Russian Federation

## Abstract

Numerous studies are devoted to the intestinal microbiota and intercellular communication maintaining homeostasis. In this regard, vesicles secreted by bacteria represent one of the most popular topics for research. For example, the outer membrane vesicles (OMVs) of *Bacteroides fragilis* play an important nutritional role with respect to other microorganisms and promote anti-inflammatory effects on immune cells. However, toxigenic *B. fragilis* (ETBF) contributes to bowel disease, even causing colon cancer. If nontoxigenic *B. fragilis* (NTBF) vesicles exert a beneficial effect on the intestine, it is likely that ETBF vesicles can be utilized for potential pathogenic implementation. To confirm this possibility, we performed comparative proteomic HPLC-MS/MS analysis of vesicles isolated from ETBF and NTBF. Furthermore, we performed, for the first time, HPLC-MS/MS and GS-MS comparative metabolomic analysis for the vesicles isolated from both strains with subsequent reconstruction of the vesicle metabolic pathways. We utilized fluxomic experiments to validate the reconstructed biochemical reaction activities and finally observed considerable difference in the vesicle proteome and metabolome profiles. Compared with NTBF OMVs, metabolic activity of ETBF OMVs provides their similarity to micro reactors that are likely to be used for long-term persistence and implementing pathogenic potential in the host.

## Introduction

Our knowledge of the microbiome has greatly expanded over the last few years. Multiple cell-cell communications between the gut microbiota and host promote homeostasis^1, 2^. Among various bacterial species, the *Bacteroides* phylum is highly represented, accounting for ten to twenty percent of the bacterial population in the colon^3^. Strong commensalism relationships that are developing between *Bacteroides* and other bacterial species today are well described^4^. For instance, *B. thetaiotaomicron*, as a saccharolytic member, harvests the gut epithelium-rich sugar mucin, making it available to species within the microbiota that lack this capability^5–7^. The sialic acid that *B. thetaiotaomicron* releases from the mucus can be catabolized by both *C. difficile* and *S. Typhimurium*, providing these strains with a growth advantage^8^. The ability of the microbiota to use sialic acid therefore depends on the action of *B. thetaiotaomicron*, and mutants that lack sialidase fail to enhance the growth of these two pathogenic bacteria^9^. Moreover, species of *Bacteroides* can elicit immune responses^10^. The most interesting member of the genus *Bacteroides*—*B. fragilis*—has been shown to play an anti-inflammatory role by acting on regulatory T (*Treg*) cells^11^. *B. fragilis* produces surface polysaccharide A (PSA), a microorganism-associated molecular pattern that is recognized by toll-like receptor 2 (*TLR2*) on *Treg* cells. The engagement of *TLR2* and PSA leads to *Treg* cell induction and limits the TH17 response, thereby promoting tolerance and immune suppression in the gut^12^. Despite the ability of immune regulation by PSA, *B. fragilis* is also an opportunistic pathogen and the most commonly isolated anaerobe from human infections such as intra-abdominal and brain abscesses^13–17^. Furthermore, toxigenic *B. fragilis* can promote intestinal inflammation and, in some cases, may contribute to colon cancer^18–21^. If most of the bacterial types have different mechanisms of virulence factor spreading or possibilities of nutrient exchange, *B. fragilis* did not show any evidence of this function^22^. Today, it is well known that gram-negative bacteria have evolved mechanisms of expressing different proteins including virulence factor delivery. Well-studied examples include type III, IV and VI secretion systems, which are required for the transport of virulence factors to the host cells^23–25^. The genomic studies of *B. fragilis* did not show evidence of type III, IV, autotransporter, or two-partner secretion systems^22^. There are genes for Hly type I secretion systems, which are similar to the hemolysin type I secretion system HlyDb of *Escherichia coli* ^26^. VI secretion system (T6SSs) was recently discovered in a few *Bacteroides* strains, thereby extending the presence of these systems beyond *Proteobacteria* ^27^. Comprehensive analysis of all sequenced human gut *Bacteroidetes* strains has shown that more than half contain T6SS loci^27^. T6SS as a multiprotein complex is specially organized into three distinct genetic architectures (GA): GA1, GA2 and GA3 loci. GA3 loci of *Bacteroides fragilis* could be a source of novel effectors and immunity proteins but not the main transport mechanism^28^. Thus, outer membrane vesicle (OMV) trafficking remains the most likely mechanism of gram-negative bacteria secretion and one of the well described for *B. fragilis* ^29, 30^. Moreover, surface-located PSA modulating the host immune system was first described as involving OMVs^31^. Recently, OMVs from *B. fragilis* and other *Bacteroides* members were found to participate in the establishment of a cooperative ecosystem in the gut^32^. In the model proposed by Rakoff-Nahoum *et al*.^33^, *Bacteroides* species secrete OMV, which can break down polysaccharides for the benefit of the other species present in the community^33^. One of the most striking characteristics of nontoxigenic *B. fragilis* OMVs is the abundance of acidic hydrolytic enzymes, mainly glycosidases and proteases, some of which were shown to be active *in vitro* ^34^. The proteolytic activity of OMVs has been demonstrated by SDS-PAGE gel electrophoresis containing gelatin following renaturation and incubation at 37 °C. The authors proposed that OMVs equipped with hydrolytic enzymes could help in securing nutrients for the benefit of the whole bacterial community present in the microbiota, uncovering a novel function for *B. fragilis* OMVs^34^. However, if the commensal *B. fragilis* OMVs function seems to be clear, then the toxigenic strain OMVs may contribute to intestinal inflammation development and have not yet been investigated. Moreover, most of the studies provide comprehensive proteome analysis of OMVs^34, 35^; however, for the first time, we prepared combined proteome and metabolome OMV data. According to our results, the biochemical reactions, represented in the OMVs of toxigenic *B. fragilis*, were prolonged due to enzyme activity after releasing of vesicles in the medium. To verify this claim we performed a comparative proteome and metabolome analysis of nontoxigenic (NTBF) and toxigenic *B. fragilis* (ETBF) OMVs and reconstructed metabolic maps of active pathways. If *B. fragilis* has special mechanisms of protein sorting to the OMVs we believe that the OMV metabolite profile depends on the OMV protein composition. Moreover, the protein and metabolic activities of ETBF may differ from those of NTBF due to its pathogenic nature. Thus, ETBF exploits OMVs as microreactors, continuing the reaction away from the cells, with substrates not only contained within but also with new substrates captured and processed from the external environment, providing virulence factor delivery and contributing to pathogen adaptation.

## Results

### Comprehensive proteome analysis of NTBF and ETBF vesicles

For both *B. fragilis* strains (ETBF and NTBF), full genome sequencing was performed. Detailed information about the alignment of the two sequenced genomes (BOB25 = ETBF and JIM10 = NTBF) can be found in Supplementary Information, Table S1-1, S1-3 and Figure S1. The procedure for vesicle isolation, purification, counting of the vesicle number and size distribution can also be found in the Supplementary Information and Figure S2.

Isolated and purified vesicles, as well as bacterial cells, of both strains independently were analyzed by HPLC-MS/MS. Analysis of the proteome of bacterial cells was performed as the combined proteomes of the membrane and cytoplasm cell fractions. SDS-PAGE analysis was provided for all samples (Figure S3). HPLC-MS/MS analysis of ETBF and NTBF OMVs was performed for three independent biological repeats with three technical repeats for each sample. HPLC-MS/MS analysis of ETBF and NTBF cells fractions was performed for two independent biological repeats with three technical repeats for each sample As a result, 49 HPLC-MS/MS runs were carried out during comprehensive proteome analysis of NTBF and ETBF vesicles and cells, and 2,430,656 MS/MS spectra were obtained. In total, 17,644 unique peptides were identified against the database constructed by *B. fragilis* BOB25 proteins (ProtDB). Additionally, 1,465 proteins were identified by 2 or more peptides across all experiments (cells and vesicles). Score thresholds and FDR are presented in Table S2. The complete list of the identified peptides and proteins are provided in Tables S3-1–S3-7 and S4, respectively. These tables contain information about the peptide sequence, PTM, PSM score, charge, m/z value, protein sequence coverage and number of identified unique peptides per protein.

**Figure 1:**
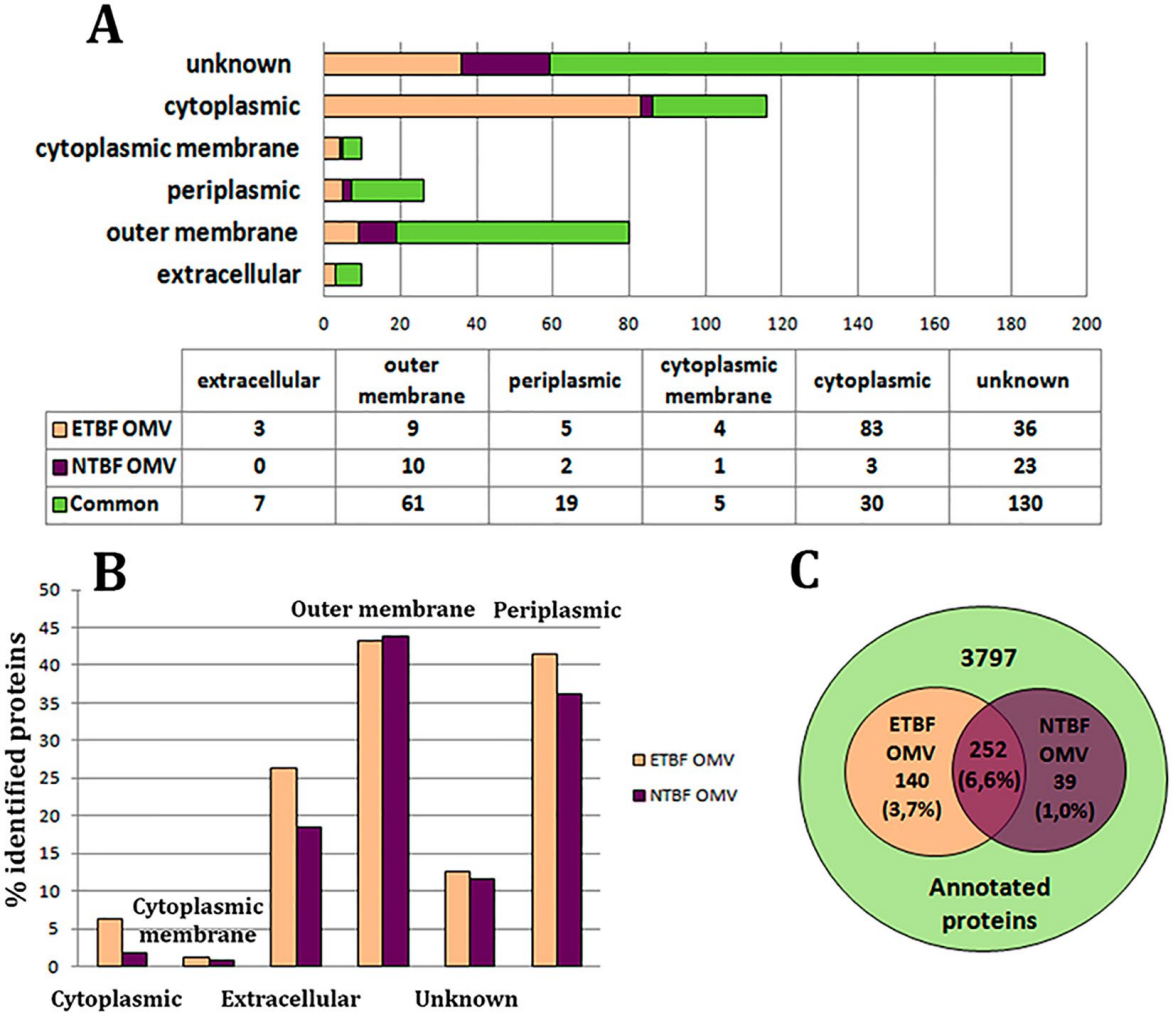
Distribution of the identified OMV proteins according to their subcellular localization. (**A**) Predicted subcellular localization of the proteins isolated from ETBF and NTBF OMVs according to PSORTb 3.0. (**B**) Percentages of the proteins identified in both types of vesicles relative to the total number annotated for *B. fragilis* proteins according to their subcellular localization. (**C**) The Venn diagram shows the comparative analysis and individual differences of the number of proteins identified in both types of vesicles relative to the total number annotated for *B. fragilis* proteins.

As a result, 823 proteins were identified in both ETBF and NTBF OMV preparations (Table S5). For further analysis, we used only 431 proteins, which were identified in at least two of the three independent preparations of *B. fragilis* OMVs. For ETBF vesicles, we identified 392 proteins, and for NTBF vesicles, we identified 291 proteins. To predict the subcellular localizations of the proteins isolated from ETBF and NTBF OMV, PSORTb 3.0 was used **(**Fig. 1A **)** ^36^.

Proteins identified in ETBF and NTBF OMVs were mostly similar (Table S5). Among the detected outer membrane proteins, we identified the group of proteins named TonB proteins. TonB-dependent receptors and TonB-linked outer membrane proteins are the part of the transporter complexes including these outer membrane proteins that are likely to import large degradation products of proteins (e.g., RagA) or carbohydrates (e.g., SusC) as nutrients^6, 37^.

As expected, we identified 19 periplasmic proteins as common for the two types of vesicles, and most of them were hydrolases. 5 identified periplasmic proteins were unique for ETBF OMVs and 2 were observed only in NTBF OMVs including 2′, 3′-cyclic nucleotide 2′-phosphodiesterase and alkaline phosphatase. Among the cytoplasmic membrane proteins of ETBF OMV, preprotein translocase subunit SecD and succinate dehydrogenase flavoprotein subunit are involved in the citrate cycle^38^. Cell division protein FtsH observed in ETBF OMV is an ATP- and Zn^2+^-dependent metalloprotease, which is anchored to the cytoplasmic membrane via two transmembrane segments in such a way that the very short amino- and the long carboxy termini are exposed to the cytoplasm^39^. For NTBF vesicles, we found only one cytoplasmic membrane protein—thiol-disulfide interchange protein, which is a protein with disulfide isomerase and oxidoreductase activity^40^. Interestingly, we identified flagellar motor protein MotA in both types of vesicles. Considering that *B. fragilis* has no flagellum, this protein may act as an H^+^ channel that allows the flux of protons outside the bacterial cell to maintain the required cellular pH and membrane charge^41^.

The essential difference among the proteins identified in the OMVs of both strains was observed for cytoplasm-located proteins (Fig. 1A and Table S5-1). Compared with NTBF, we identified components of the RNA translation system, including elongation factor G, Ts, Tu, translation initiation factor IF-2 and multiple ribosomal proteins in ETBF OMV. Since the ribosomal proteins detection in OMVs preparation could be the result of contamination by cell debris, additional step of sucrose purification was added before proteome analysis (described in Supplementary Information). Resulted proteomic data obtained by using two protocols (with or without sucrose purification) were compared. As expected, we didn’t observed most of ribosomal proteins and elongation factors but otherwise the proteomic profile remained unchanged (Supplementary Table S5-6). Only 50 S ribosomal protein L7/L12 was detected in NTBF OMV. Moreover, we identified the proteins involved in DNA replication and transcription. According to our results, NTBF OMVs contained only two components of the RNA translation system—elongation factor G and Tu—and did not comprise any proteins involved in DNA replication or transcription. Additionally, the identified transcription and translation components included several molecular chaperones involved in the process of RNA degradation that were common for the two types of vesicles (Table S5-2). Several stress-induced proteins, such as pirin, non-heme ferritin, thioredoxins and phosphate starvation protein PhoH, were also observed among the cytoplasm-located proteins of ETBF OMVs.

Clostripain is one of the unique proteins identified in both types of vesicles. According to a recent publication, Clostripain or Fragipain was described as the protein contributing to fragilysin toxin maturation^42^. NTBF also contains clostripain, but it is not active because of an insertion mutation of the *clostripain* gene^43^. According to our sequencing data of the NTBF (JIM10) *clostripain* gene, it contains an amino acid mutation (Q/L) at the C-end of the protein, possibly modulating its activity. The results of quantitative proteome analysis are shown in Table 1.

**Table 1:** Comparative proteomic analysis of proteins identified in ETBF and NTBF OMVs.

Gene	Protein Name	Adjusted p value	MV (emPaI)ETBF/MV(emPaI)NTBF	Fold change
VU15_RS07335	membrane protein	0,00342588	0,02536178	5,3
VU15_RS07330	membrane protein	0,0144819	0,11667014	3,1
VU15_RS13200	carbohydrate-binding protein	0,02147599	0,20806557	2,3

Mean value (MV) of emPaI(ETBF) and emPaI(NTBF) indexes were calculated for three biological repeats. Fold change is the log_2_ of MV of emPaI(ETBF) and emPaI(NTBF).

### Virulence factors associated with OMVs

**Figure 2:**
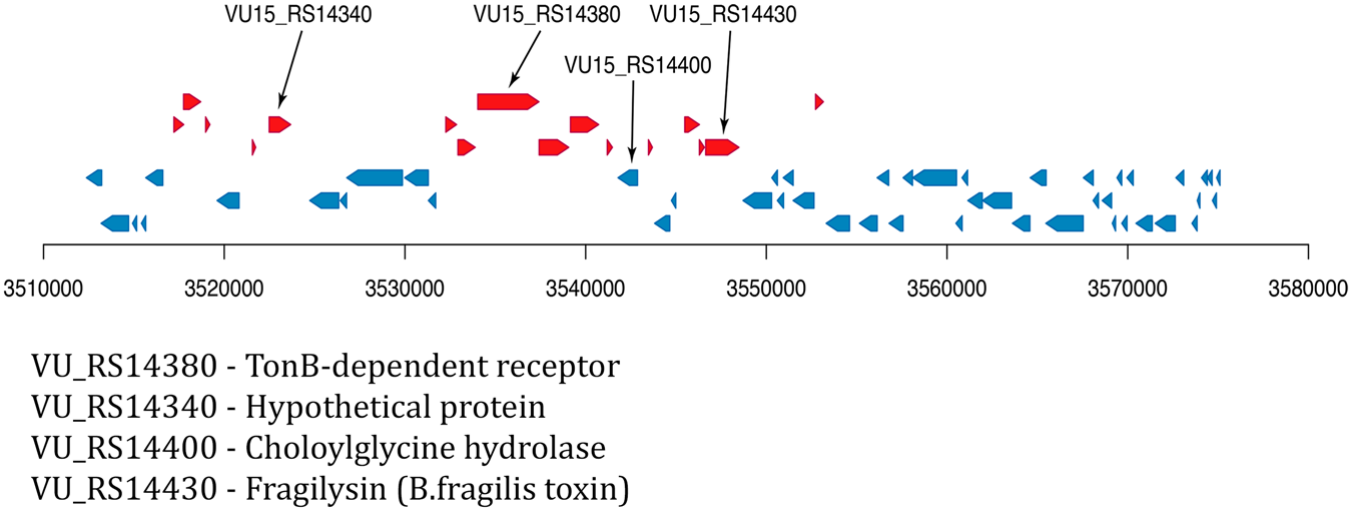
Pathogenicity island of the BOB25 strain. Red and blue arrows indicate genes coding potential virulence factors. Black arrows indicate the virulence factors, identified in ETBF OMVs.

According to the genome data, *B. fragilis* contains a pathogenicity island (BfPAI) located within the conjugative transposon CTn86 of 63,282 bp^44^. After accurate analysis of the ETBF OMV proteome, we have found several proteins belonging to the pathogenicity island that could be delivered to the host cells via OMVs: TonB-dependent receptor (VU15_RS14380), hypothetical protein (VU15_RS14430), which is potentially outer membrane protein OmpA according to homology analysis provided by protein blast, and choloylglycine hydrolase (VU15_RS14400) (Fig. 2 and Table S1-3). Interestingly, choloylglycine hydrolase has been previously found to show special activity against the taurine conjugates of dihydroxy bile acids^45^. Enterotoxin-fragylisin (VU15_RS14340) was also found to be transported by the vesicles in our previous publication^46^. According results of proteogenomic analysis we determined more potential virulence factors which could be essential for ETBF pathogenity. But these data should be clarified since they can be the result of sequencing errors and subsequent annotations, respectively (Table S1-2 and Figure S4).

### Enzymes and functional metabolic pathways

**Figure 3:**
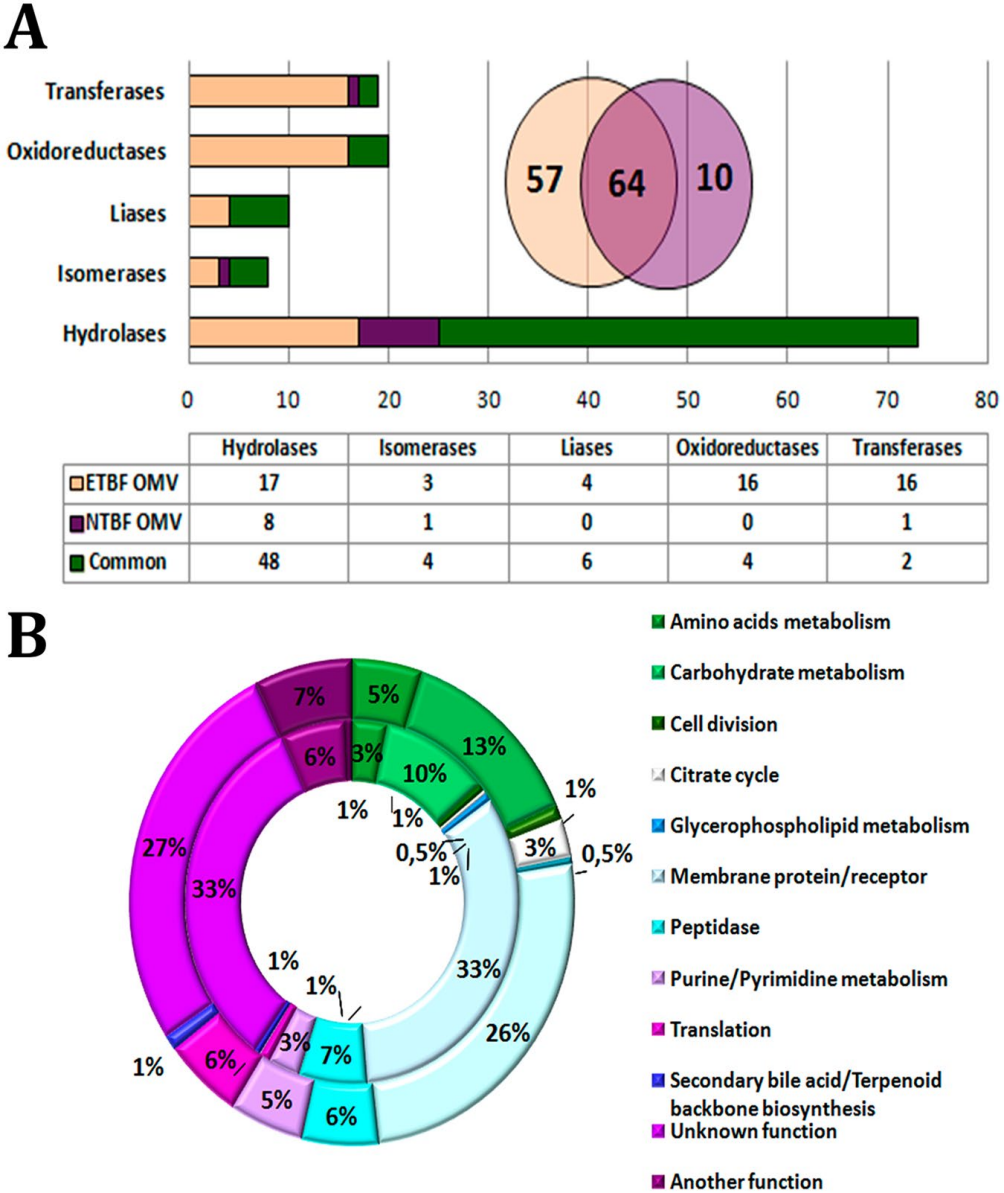
Enzymes identified in both types of vesicles and functional metabolic pathways. (**A**) The columnar histogram represents distribution of enzymes identified in both types of vesicles according to enzyme class. The Venn diagram represents comparative analysis of the total number of enzymes identified in both types of vesicles. (**B**) The main pathways and functional groups of proteins identified in both types of vesicles. External histogram—the proteins identified in ETBF OMVs; internal histogram—the proteins identified in NTBF OMV. The number of identified proteins involved in a particular metabolic pathway is represented as the percentage of the total number of proteins identified in ETBF or NTBF OMVs.

As a result of comparative proteome analysis for both types of vesicles (NTBF OMVs and ETBF OMVs), we identified 5 classes of enzymes: hydrolases, isomerases, lyases, oxidoreductases, and transferases (Fig. 3A and Table S5-3).

ETBF OMVs are characterized by a larger number of enzymes than NTBF OMVs. The total protein number in ETBF OMVs is 392, of which 121(31%) are enzymes. Whereas among the 291 proteins found in NTBF OMVs, 74(25%) are enzymes. NTBF OMVs contain a substantial number of hydrolases (76%) compared with ETBF OMV (54%) (Fig. 3A and Table S5-3). The numbers of identified isomerases (n_(ETBF OMV)_ = 7 and n_(NTBF OMV)_ = 5) and lyases (n_(ETBF OMV)_ = 10 and n_(NTBF OMV)_ = 6) were approximately equally distributed between the two types of vesicles (Fig. 3A). Additionally, we identified a substantial number of transferases(n_(ETBF OMV)_ = 18 and n_(NTBF OMV)_ = 3) and oxidoreductases (n_(ETBF OMV)_ = 20 and n_(NTBF OMV)_ = 4), including glutathione peroxidase in ETBF OMVs (Fig. 3A). According to our data, most of the ETBF and NTBF OMV enzymes were associated with carbohydrate metabolism, glycan degradation and amino acid and nucleotide sugar metabolism (Fig. 3B). Additionally, if hydrolases providing a nutrition function compose the majority of NTBF OMV enzymes involved in carbohydrate metabolism, then the enzymes detected in ETBF OMVs are involved in basic energy-enriching metabolic pathways including glycolysis, the pentose phosphate pathway and TCA cycle. Moreover, both types of vesicles equally compose the proteins supporting amino acid and purine/pyrimidine metabolism.

**Figure 4:**
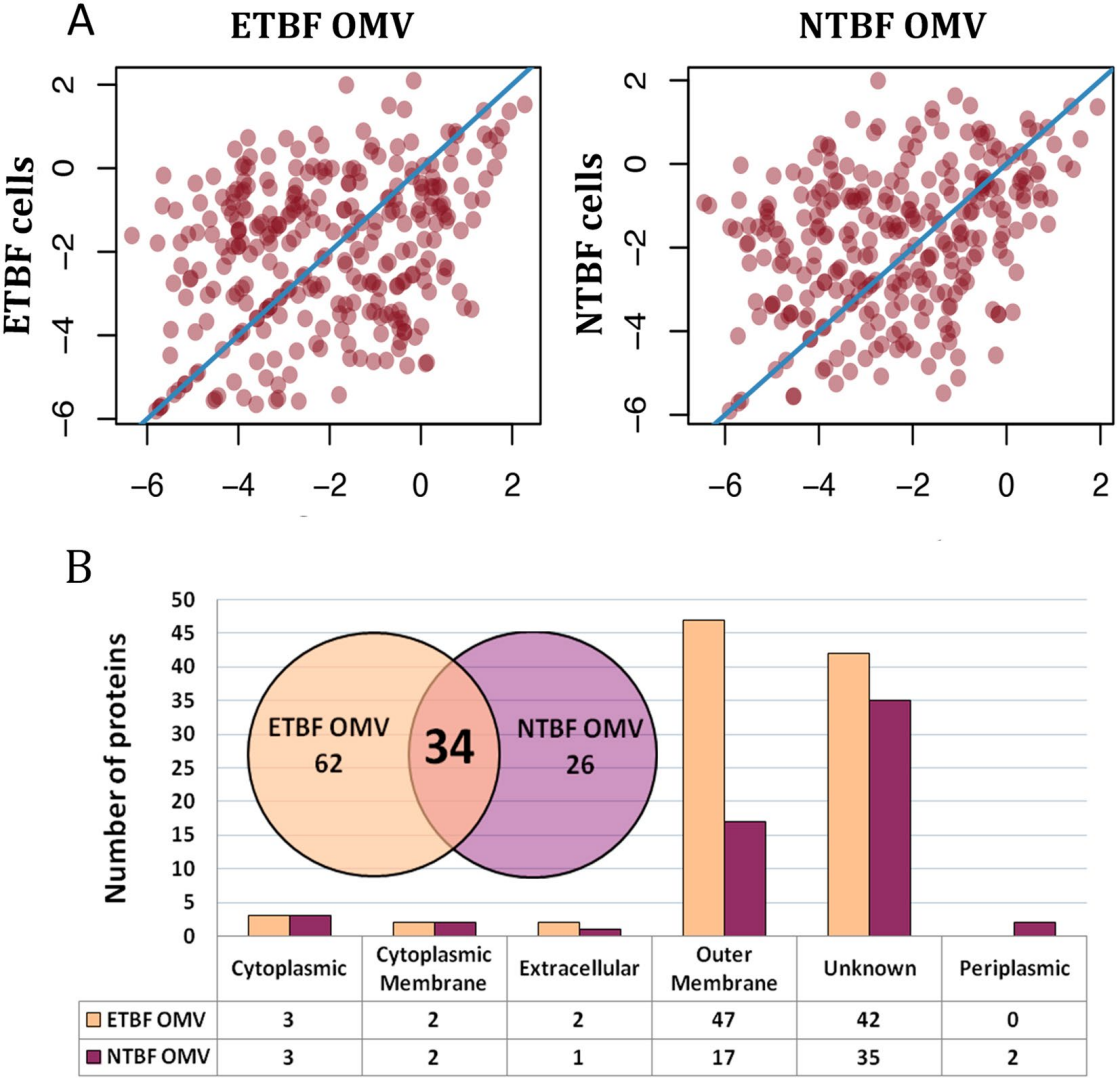
Comparative analyses of ETBF and NTBF proteins identified in *B. fragilis* cells and OMVs. (**A**) Correlation analysis of the representation of the proteins identified in ETBF/NTBF cells vs. ETBF/NTBF OMVs. Each point indicates emPAI calculated for the proteins identified in both ETBF/NTBF cells and ETBF/NTBF OMVs. (**B**) Proteins identified exclusively (the proteins were not identified in cells proteome) in ETBF/NTBF OMVs. The columnar histogram shows ETBF/NTBF OMV protein distribution according to subcellular localization. The Venn diagram describes individual differences in the total number of proteins identified exclusively (the proteins were not identified in cells proteome) in ETBF/NTBF OMVs.

To determine the probable protein sorting contributing to the well-defined set of proteins for both types of OMVs, we compared the relative representations of OMVs and bacterial cell proteins for both *B. fragilis* strains **(**Tables S3-1–S3-7, S4 and S5-5 **)**. As expected, there was no correlation of the protein relative representation between the vesicles and cell proteomes (Fig. 4). We identified an increase of the different proteins amounts of both types of OMVs, which were represented as the minor part of the cell proteins, suggesting that there is a special sorting mechanism that contributes to the individual differences between the two types of vesicles. For ETBF OMVs, we identified 96 proteins that were not detected in ETBF cells, including chitobiase, serine protease, several TonB-dependent receptors and others. We observed the same phenomena comparing NTBF OMVs and NTBF cells (Table S5-5).

### Comprehensive metabolome analysis of NTBF and ETBF vesicles

**Figure 5:**
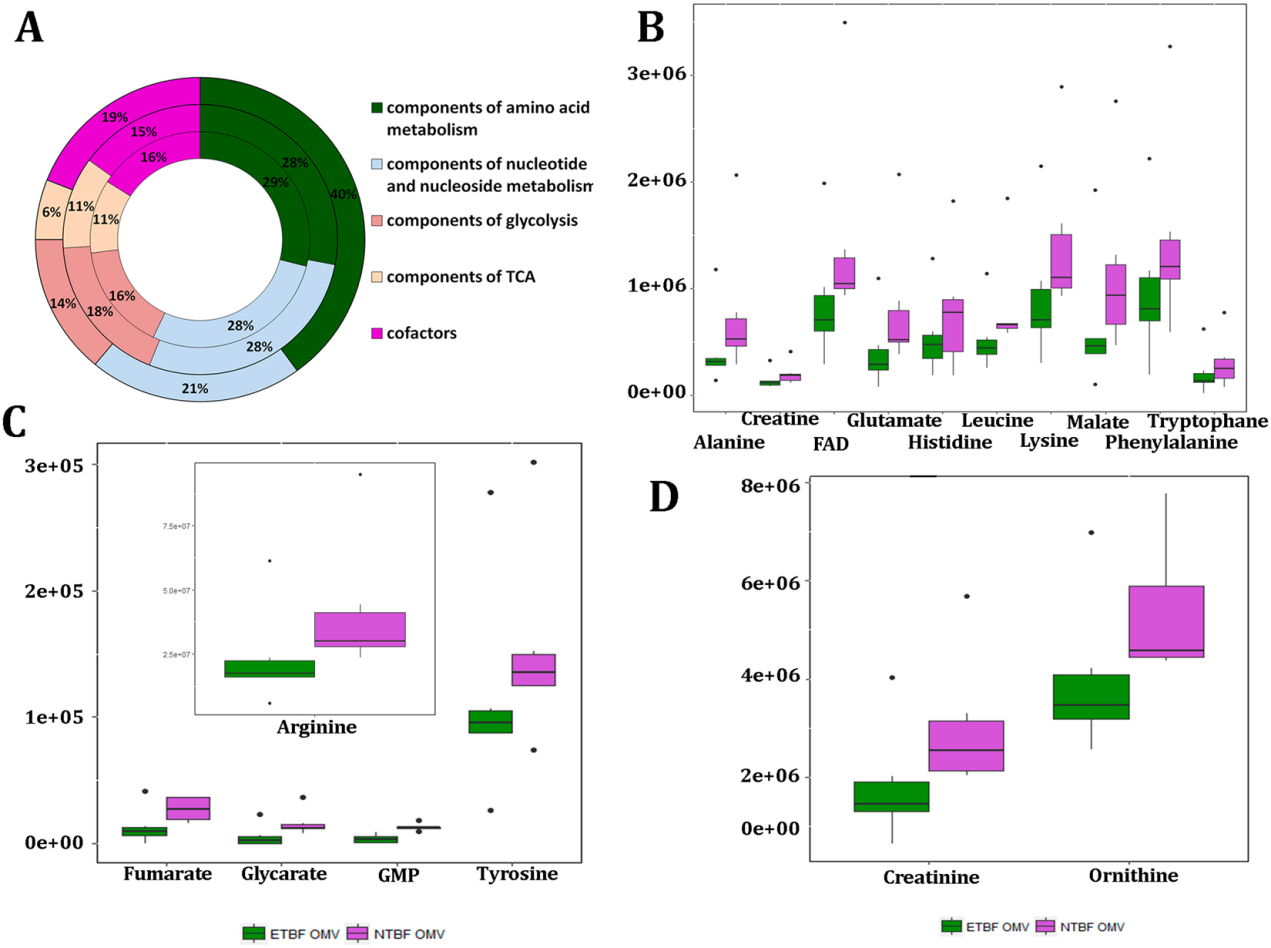
Qualitative and quantitative analyses of ETBF and NTBF metabolites identified in *B. fragilis* cells and OMVs. (**A**) Qualitative analysis of metabolites identified in OMVs (external histogram), ETBF cells (middle histogram) and NTBF cells (internal histogram). Metabolites involved in certain metabolic pathways are represented as percentages of the total number of identified in ETBF OMVs/cells and NTBF OMVs/cells metabolites. (**B**–**D)** The Box plots show the quantitative differences of the amounts of several metabolites identified in ETBF and NTBF OMVs.

Using metabolite standards, we performed HPLC-MS/MS analysis of 95 metabolites that are highly represented in bacterial cells (Table S6) and supplemented it by GS-MS analysis. In particular, we detected the components of amino acid metabolism, nucleotide and nucleoside metabolism, glycolysis, the TCA cycle and several cofactors (Fig. 5A and Table S6-1). The metabolic analysis for the bacterial cells of both strains compared with OMVs was also performed (Table S6-1). Moreover, according to our GC-MS data, both types of OMVs contain the oxidized and reduced forms of free fatty acids (Table S6-4).

Comparative quantitative metabolomic analyses (Tables 2 and S6-2) showed the decrease of the metabolites amount in ETBF vesicles compared with those in NTBF, indicating the existence of active metabolic pathways (Fig. 5B–D). Importantly, we identified 6 essential amino acids that were increased in NTBF vesicles against ETBF OMVs. Creatinine and creatine, glycerate-2P, components of the TCA cycle (fumarate and malate), cofactor FAD and GMP were increased in NTBF OMVs (Table 2). We detected D-glycerate-2-P accumulation in NTBF OMVs, indicating the presence of glycolysis gaps and partial activity of this pathway. The detected decrease in the TCA cycle intermediates and specific cofactor FAD could be the result of cycle activity in ETBF vesicles. We identified increasing GMP amounts in NTBF vesicles, which could be the result of the RNA degradation process followed by purine nucleotide catabolism in NTBF vesicles, as confirmed by specific enzymatic activity in vesicles.

**Table 2:** Results of quantitative analysis of metabolic compounds of OMVs isolated from ETBF and NTBF.

ID	Compound name	Fold (NTBF/ETBF)	Retention time	P-value	Q-value
C00082	L-Tyrosine	1.3	6.491988	0.001294	0.032347
C00047	L-Lysine	1.6	4.06667	0.009666	0.120819
C00123	L-Leucine/L-Isoleucine	1.6	3.375899	0.019762	0.141159
C00078	L-Tryptophane	1.5	3.166178	0.024922	0.10384
C00079	L-Phenylalanine	1.5	3.038711	0.028793	0.102833
C00183	L-Valine	1.7	2.887239	0.034297	0.107178
C00025	L-Glutamate	2.0	2.799374	0.038022	0.111829
C00135	L-Histidine	1.4	2.692345	0.043177	0.119937
C00041	L-Alanine	1.8	2.543114	0.050697	0.129244
C00077	L-Ornithine	1.3	3.232747	0.023135	0.115675
C00062	L-Arginine	1.8	4.00147	0.010308	0.103084
C00197	D-Glycerate-2-P	2.8	8.069242	0.000473	0.023669
C00122	Fumarate	2.6	3.373054	0.019824	0.123898
C00149	Malate	1.8	3.182635	0.024466	0.111209
C00016	FAD	1.7	2.887239	0.034297	0.114324
C00144	GMP	3.6	4.204983	0.008449	0.140815
C00791	Creatinine	1.7	3.611015	0.015364	0.128033
C00300	Creatine	1.4	3.046563	0.028536	0.109754

**Figure 6:**
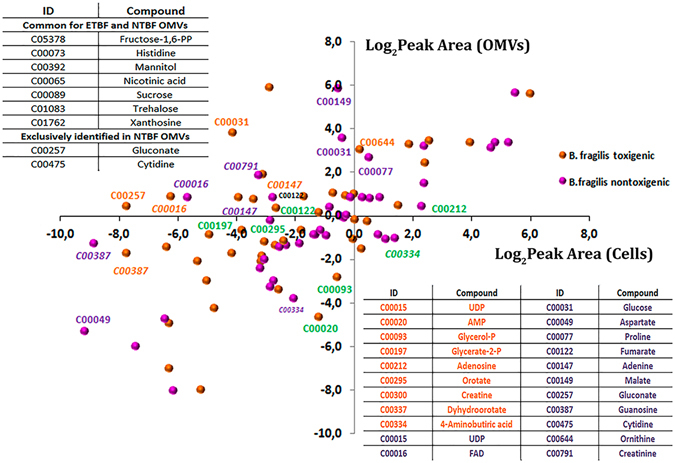
Comparative analysis of the relative representation of metabolites in cells and vesicles. Correlation analysis of the representation of the metabolites identified in ETBF/NTBF OMVs and cells. Metabolites are indicated by orange (identified in ETBF OMVs) or pink (identified in NTBF OMVs) circles. The coordinates of each circle indicate the mean of the logarithmic peak area calculated for metabolites identified in OMVs and cells, respectively. Metabolites highly represented in ETBF OMVs compared with ETBF cells are signed in orange color. Metabolites highly represented in NTBF OMVs compared with NTBF cells are signed in purple color. Metabolites highly represented in ETBF/NTBF cell compared with ETBF/NTBF OMVs are signed in green color (according Table S6-2). The table located in the upper left corner contains metabolites that were not detected in cells but only in OMVs. The table in the right bottom contains the decryption of the KEGG nomenclature. The full data represented in Table S6.

Metabolites may also be trapped in the vesicles as well as the proteins. To verify it, we compared the result of the metabolome analyses of ETBF and NTBF cells and OMV **(**Table S6-2 **)**. As expected, we observed a weak cell-OMV metabolic correlation **(**Fig. 6). The following metabolites were not detected in the cells and were highly represented in both types of OMVs: L-histidine, mannitol, nicotinic acid, sucrose, xanthosine, D-fructose-1,6-PP, and trehalose. We also identified unique metabolites exclusively represented in NTBF OMVs: cytidine and gluconate. Compared to the OMV metabolite composition, *B. fragilis* cells were mostly similar, but several metabolites were detected exclusively in ETBF or NTBF cells **(**Fig. 6 **)**.

### ETBF and NTBF OMV metabolism reconstruction

**Figure 7:**
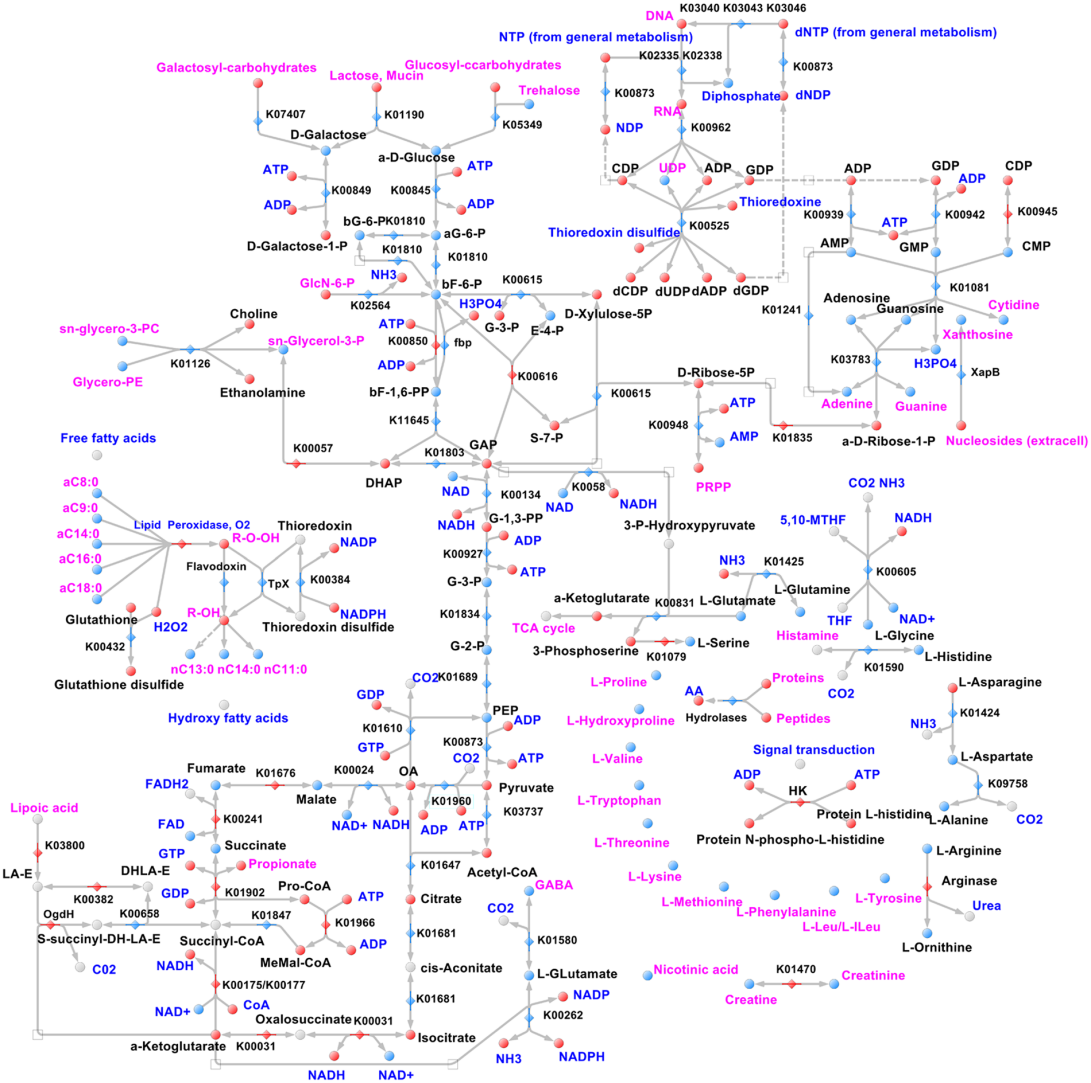
Reconstructed metabolic maps of ETBF OMVs. Metabolites that are identified/not identified are colored in blue/red spheres. The blue/red rhombus indicates identified/not identified enzymes. The main reconstructed pathways are shown in the picture. sn-glycero-3-phosphocholine- sn-glycero-3-PC; Glycerophosphoethanolamine-Glycero-3-PE; bG-6-Pb-D-Glucose-6-phosphate; aG-6-Pa-D-Glucose-6-phosphate; bF-6b-D-Fructose-6-phosphate; E-4-PD-Erythrose-4-phosphate; GAPD-Glyceraldehyde-3-phosphate; G-3-PD-Glycerate-3-phosphate; G-2-PD-Glycerate-2-phosphate; G-1,3-PPD-Glycerate-1,3-bisphosphate; dF-1,6-PPb-D-Fructose-6-bisphosphate; S-7-P - D-Sedoheptulose-7-phosphate, dihydrolipoamide E - DHLA-ELipoamide-E -LA-ES-succinyl-DHLA-E - S-succinyl-dihydrolipoamide-EPropanyl-CoA - Pro-CoAMethylMalonyl - CoA - MeMal-CoAOxaloacetate - OAPhosphocholine - PCPhosphoethanolamine - PE5,10-Methylene-THF - 5,10-MTHFL-Leucine/L-Isoleucine - L-Leu/L-ILeuHistidine kinase –HK.

**Figure 8:**
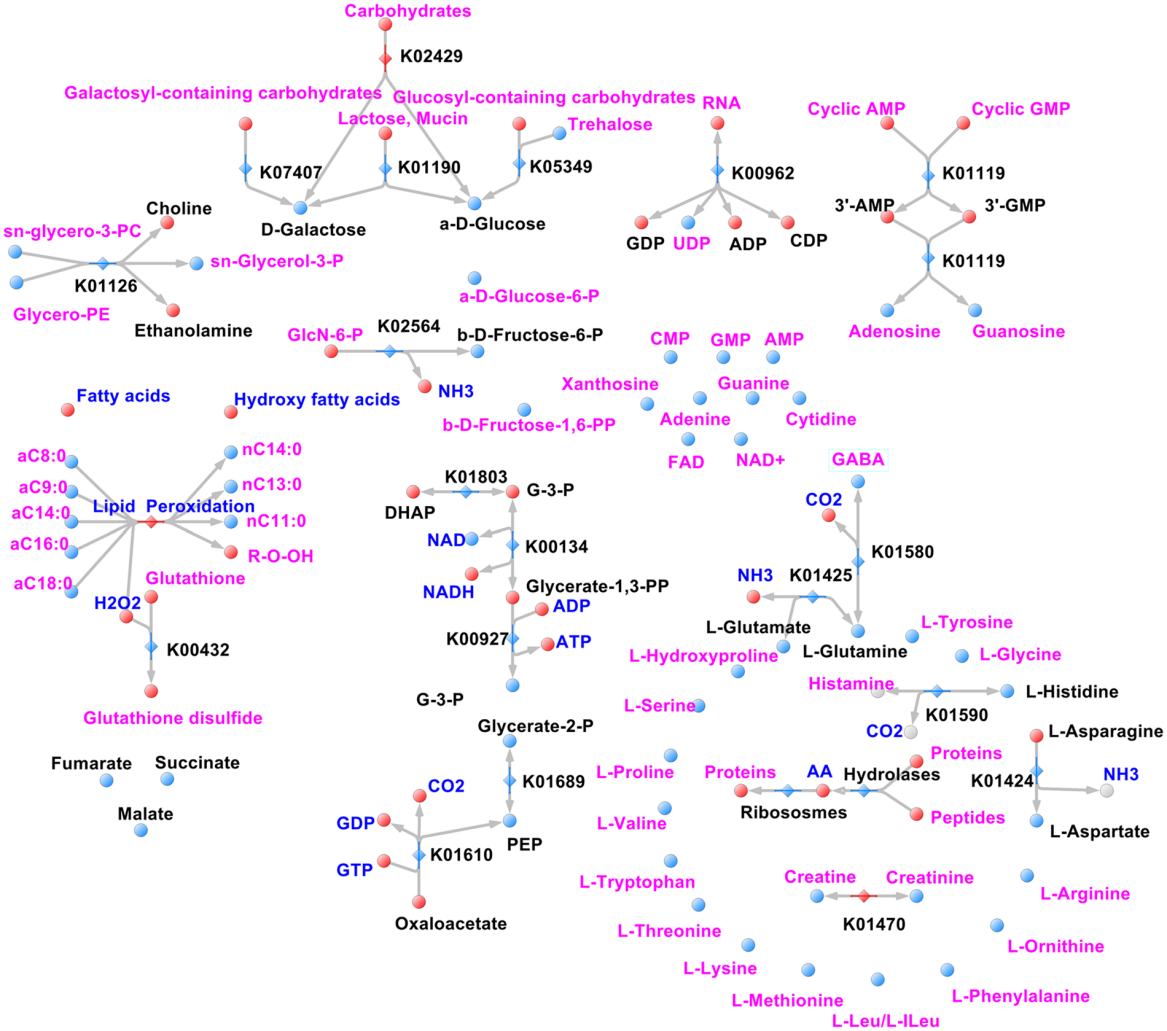
Reconstructed metabolic maps of NTBF OMVs. Metabolites that are identified/not identified are colored in blue/red spheres. The blue/red rhombus indicates the identified/not identified enzymes. The main reconstructed pathways are shown in the picture.

To build comprehensive metabolic networks for OMV metabolism, we used the proteomic and metabolomic data combined with proteogenomic annotation data provided previously and developed in our laboratory using the metabolic visualization software “Pathways editor” (unpublished). For metabolites that could participate in non-annotated *B. fragilis* reactions (for example, arginine/ornithine transformation), we hypothesized the proteins that could carry out these reactions. We have mapped the enzyme on the map when the product of the reaction was detected. Finally, we obtained a full metabolic map of ETBF and NTBF OMVs, including different interrelated metabolic pathways, as confirmed by HPLC-MS/MS and GC-MS data. All of the detected intermediates and cofactors of the reconstructed reactions, as well as the interrelation of each pathway, were indicated in the final metabolic maps **(**Figs 7 and 8).

Thus, metabolic reconstruction for ETBF OMVs contained major metabolic pathways, such as glycolysis, the pentose phosphate pathway, the TCA cycle, amino acid metabolism, purine and pyrimidine metabolism, including active DNA and RNA synthesis and the lipid peroxidation defense mechanism. The most filled pathways in the reconstructed map of ETBF OMVs were glycolysis and the TCA cycle. We detected several metabolites formed in the TCA cycle: malate, succinate, fumarate and cofactor FAD, as well as pyruvate carboxylase and phosphoenolpyruvate carboxykinase enzymes, combining glycolysis with the TCA cycle. Moreover, we identified the most important enzymes involved in the rate-limiting stage of the TCA cycle (citrate synthesis): citrate synthase, malate dehydrogenase and aconitate hydratase. Such metabolic representation of the TCA cycle and glycolysis enzymes and metabolites in OMVs indicates active energy production in vesicles. We detected nucleotide and nucleoside metabolism enzymes in OMVs, including polynucleotide phosphorylase, 5′-nucleotidase and NDP-reductase; however, in the OMV metabolome, we identified only UDP as a single accumulation point. Along with these findings, we detected AMP, CMP and GMP and their metabolism products: adenosine, guanosine, xanthosine, cytidine, adenine and guanine. Simultaneously, pyrimidine metabolism (purine and cytidine) is active as marked on the map.

Based on the obtained proteo-metabolomic data, we reconstructed the lipid peroxidation defense pathway for ETBF OMVs, combining glutathione peroxidation and thioredoxin redox systems. In addition to the detected free long-chain fatty acids, we observed the hydroxyl derivative of fatty acids in the OMV metabolome, which could indicate the activity of membrane lipid peroxidation defense processes in OMVs.

Despite the detection of all 20 amino acids in ETBF OMVs, we have not identified most enzymes involved in amino acid metabolism, excluding several enzymes, catalyzing amino acid transformation. Among them, we identified the enzyme catalyzing histidine decarboxylation to histamine. Histamine is a well-investigated intermediate of histidine metabolism acting as a neurotransmitter in human organisms and is involved in local immune responses as well as the regulation of physiological function in the human intestine^47, 48^. Another interesting finding was γ-aminobutyric acid (GABA) and its biosynthesis intermediates α-ketoglutarate and glutamate detection. GABA is the main inhibitory neurotransmitter in the mammalian central nervous system. Detected GABA biosynthesis components in ETBF OMVs indicate the OMV transmission role in the microbiota-host interaction, via modulating the host’s neurophysiological and immunological response as well as previously described histamine production^49^.

NTBF OMV metabolome and proteome do not include the components of main pathways. Despite the lack of meaningful enzymes in NTBF OMVs, we detected the same composition of amino acids, including essential, monophospho-nucleotides and the products of their degradation, confirming the described *B. fragilis* nutrition function. Moreover, during metabolic reconstruction, we detected the pathway with “enzymatic gaps”—the absence of one or more enzymes in the reconstructed metabolic pathway in the presence of most of its enzymes. To fill such gaps in reconstruction and identify novel protein-coding genes, we performed proteogenomic analysis. Thus, we successfully detected and marked on the map fructose-1,6-bisphosphatase (*fbp*), catalyzing phosphate cleavage from fructose-1,6-bisphosphate in glycolysis. Using the described approach, we identified the following enzymes in *B. fragilis* OMVs: histidine kinase, nucleoside (xanthosine) permease and flavodoxin. Previously, we detected xanthosine in the NTBF and ETBF OMV metabolome, but there were no xanthosine precursors and specific catabolic enzymes for xanthosine biosynthesis. Xanthosine permease (XapB), identified by proteogenomic analysis, successfully solved this problem because it catalyzed xanthosine import into the OMVs from the media. Histidine kinase detected and mapped on the reconstructed metabolic map plays a major role in signal transduction in prokaryotes and is needed for the cellular adaptation to environmental conditions and stresses^50^. We have also identified the equally important stress adaptation enzyme—flavodoxin—known as an important contributor to metronidazole *B. fragilis* sensitivity^51^.

### Fluxomic experiments

**Figure 9:**
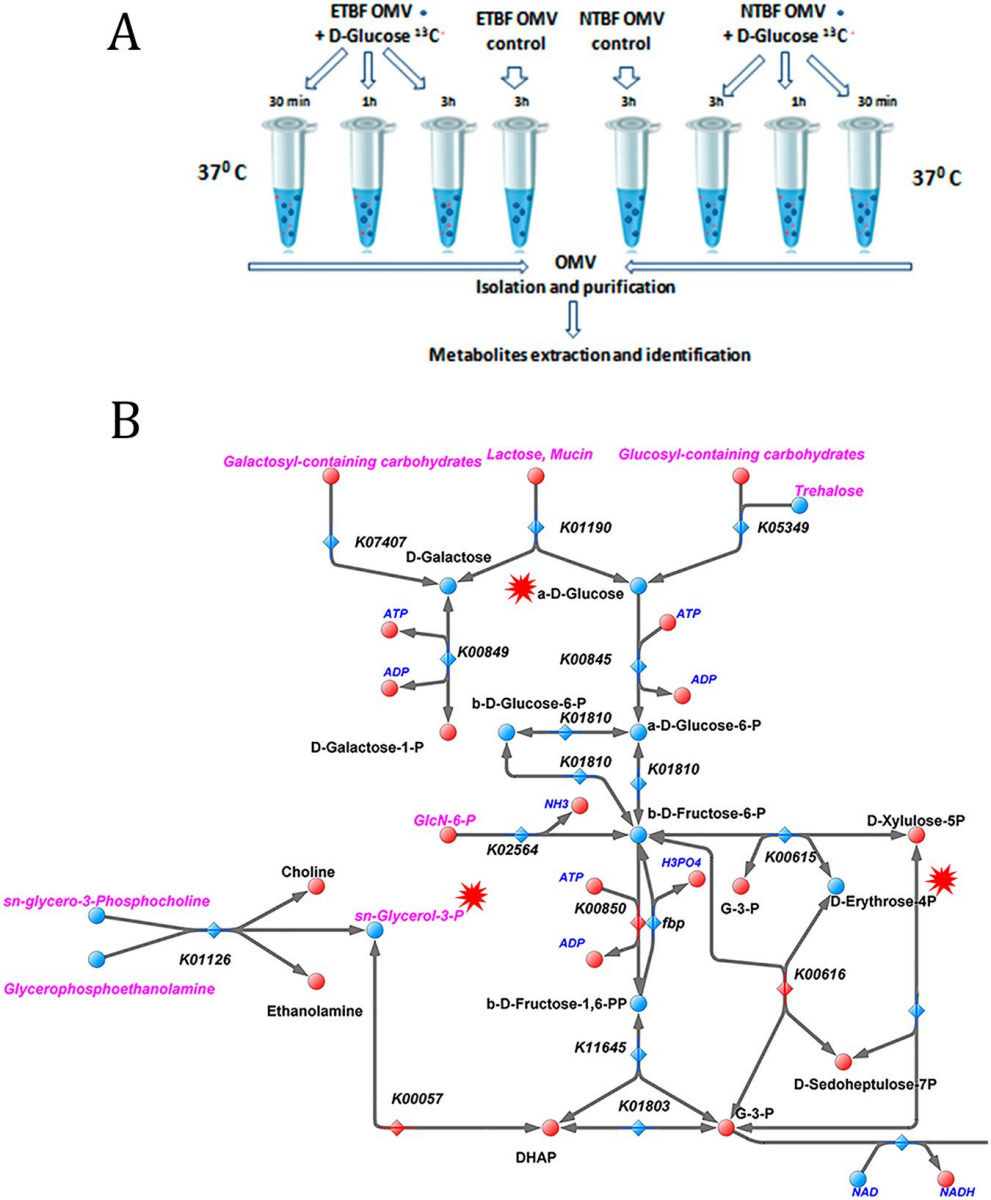
Fluxome experiment. (**A**) The main stages of the fluxome experiment include OMV incubation with isotope-labeled glucose with the following OMV isolation and purification. Extracted metabolites are utilized for metabolome analysis. (**B**) The reconstructed map of the ETBF OMV metabolic pathway (glycolysis). Labeled glucose metabolites such as ^13^C_4_-D-erythrose-4P and ^13^C_3_-Glycerol-3P were detected only in ETBF OMVs. ^13^C_6_-D-Glucose and the products of ^13^C_6_-D-Glucose catabolism, ^13^C_4_-D-erythrose-4P and ^13^C_3_-Glycerol-3P are indicated by red stars.

To test carbohydrate transporters and metabolic enzymes activity in both types of OMVs, we used fluxomic experiments with isotope-labeled glucose (^13^C_6_-D-glucose) as the substrate, supplemented with the dilution buffer. We incubated both types of OMVs and isotope-labeled glucose (^13^C_6_-D-Glucose) containing solution at 37 °C for 30 min, 1 and 3 hours. As a result, labeled glucose metabolites such as ^13^C_4_-D-erythrose-4P and ^13^C_3_-Glycerol-3P were detected only in ETBF OMV. D-Erythrose-4-phosphate is a unique pentose phosphate pathway intermediate, participating in the formation of D-fructose-6-phosphate. Glycerol-P-^13^C is usually used by bacterial cells for lipid synthesis and could be produced by the dehydrogenation of D-glyceraldehyde-3-phosphate. We detected a stable decrease of D-glyceraldehyde-3-phosphate at the third hour of incubation in ETBF OMVs, indicating the end of the metabolic process. During metabolic map reconstruction, we included the reaction of D-fructose-6-P phosphorylation by *pfkA* that was not identified but was obviously active according to HPLC-MS/MS fluxomic data. We have not observed any metabolic activity in NTBF OMVs (Fig. 9).

## Discussion

Today, there are several hypothetical models of OMV formation and mechanisms of protein sorting^52^. However, thus far, there is no exact data about the mechanism of the protein packing in OMVs. Moreover previously it has been shown, that gram negative bacteria can form two types of vesicles, consist of single and double membranes. Double membranes vesicles contain cytoplasm and cytoplasmic membrane proteins^53–55^. We also observed this phenomenon in Bacteroides fragilis (Figure S5) and after all during ETBF and NTBF OMVs proteome analysis we found that the main difference was the presence of large number of cytoplasmic proteins in ETBF OMVs. We also observed ribosomal proteins but during sucrose gradient treatment we have lost most of them, confirming that its existance can be the result of the contamination by cell debris. But we have detected ribosomal proteins only in ETBF OMVs but not in NTBF OMVs before using protocol with sucrose gradient. Given the fact that ribosomal proteins are the major part of the cellular proteins we suggest that its detected in ETBF OMVs can be the result not only contamination by cell debris but the existence of different types of OMVs (including single and double membrane OMVs) which possible can be lost during sucrose gradient purification.

Among the identified cellular proteins of ETBF OMV, there are a large number of enzymes, which contribute to hydrolase activity. Species from the genus *Bacteroides* carry multiple systems to bind to the epithelium and degrade surface located polysaccharides^30^. *B. fragilis* is also involved in charity towards other bacterial types deprived of the polysaccharide utilization opportunity. In addition to proteomic results obtained in our experiments, hydrolase and peptidase activity were confirmed by metabolomic data where we have detected high represented essential amino acids and non-phosphorylated carbohydrates. Another class of identified enzymes was oxidoreductases. Among the identified oxidoreductases, the components of oxidative stress defense mechanism (glutathione peroxidase and lipid hydroperoxide peroxidase) were the most important. Glutathione peroxidase requires adapting to the oxidative stress that accompanies an inflammatory response^56^. Several stress-induced proteins, including pirin, non-heme ferritin, thioredoxins and others, were also observed. Pirin, which was found exclusively in ETBF OMVs is stress induced in *Cyanobacteria* and may act as a quercetinase in *Escherichia coli*, the functions of pirin orthologs in prokaryotes remain mostly uncharacterized^57^. Non-heme ferritin, identified in ETBF OMV has a special oxidoreductase activity, binding ferric iron providing cellular iron ion homeostasis^58^. Thioredoxins are proteins that act as antioxidants by facilitating the reduction of other proteins by cysteine thiol-disulfide exchange^59^. PhoH is activated in response to phosphate limitation^60^. Transcriptome analysis revealed that the deletion of *phoB* affected the expression of 585 genes (more than 4-fold change) in *B. fragilis*, which included genes for stress response (chaperons and heat shock proteins), virulence (capsular polysaccharide biosynthesis) and phosphate metabolism^61^. Vesicles production increases under stress conditions^62^. Previously it has been shown that *B. fragilis* treated with bile salts overproduces fimbria and OMVs. Moreover bile salts enhance bacterial co-aggregation, bacterial-intestinal epithelial cell adhesion, biofilm formation and antimicrobial resistance of *B. fragilis* ^63^. Possible OMVs is important mechanism involved in biofilm formation as it was previously described for *Xylella fastidiosa* ^64^. The increase in OMV production containing multiple oxidoreductases may be also the stress adaptation mechanism at the expense of increasing the protein yield and promoting the distal neutralization of adverse environmental factors. We believe that secreted OMVs may surround bacteria and protect them from stress similar to a “shield” helping to adapt to the host immune reaction during infection.

OMV is also a mechanism for various virulence factors, especially toxin delivery. Some examples of OMV-associated toxins include: Shiga toxin from *Shigella dysenteriae*, LT from enterotoxigenic *E. coli*, leukitoxin from *Actinobacillus actinomycetemcomitans*, Apx toxin from *Actinobacillus pleuropneumoniae* and the best known toxin of *Helicabacter pylori*—VacA, and one of the last finding - toxins and antitoxins of *Xylella fastidiosa* OMVs^64–68^. Among the identified ETBF OMV proteins, we detected several virulence factors produced from the bacterial pathogenicity island including TonB-dependent receptor, hypothetical protein that, according to homology analysis (protein blast), belong to the membrane protein OmpA and choloylglycine hydrolase. In our recent study, we detected the major virulence factor - Fragilysin associated with OMVs^46^. Fragilysin is a *Bacteroides fragilis* toxin (*bft2*) that promotes colonic injury and inflammation through E-cadherin cleavage^69, 70^. Until recently, there was no information of possible proteases that can provide fragilysin maturation. Clostripain-cysteine protease was found as a main factor that contributed to toxin maturation^42, 43^. An unexpected result was to the identification of clostripain in OMVs, suggesting possible toxin maturation carried out in vesicles. Patatin was another potential virulence factor that was observed in OMVs. Patatin in ETBF OMVs does not belong to the pathogenicity island but is a potential virulence factor. First described as a plant storage glycoprotein with lipid acyl hydrolase activity, patatin was found in the *Toxoplasma gondii* genome and was associated with host-microbe interactions in bacteria^71^. The latter study has shown that ubiquitin, which can signal for protein degradation via the proteasome in eukaryotic cells, activates patatin-like phospholipases from *Pseudomonas aeruginosa* ^72^. Considering that patatin presumably has lipolytic activity, intracellular patatin activated by host ubiquitin may dramatically interact with lipids contained in eukaryotic cell organelles.

In addition to the proteome research of OMVs, for the first time, we performed metabolomic assay of ETBF and NTBF OMVs; according to combined proteomic and metabolomic data, we reconstructed metabolic pathways for both types of vesicles. During comparative metabolome analysis of bacterial cells and OMVs, we detected the inverse correlation between metabolites represented in cells and OMVs. We hypothesized that biochemical processes in vesicles are still active after secretion. After the detailed reconstruction of metabolic maps, we determined the huge difference between the metabolic activities of the two types of vesicles. Metabolic pathways (glycolysis, the pentose phosphate pathway, the TCA cycle and nucleotide and nucleoside metabolism) reconstructed in ETBF OMVs were fully represented in ETBF OMVs in contrast to the NTBF vesicles. We carried out the fluxomic experiments using isotope-labeled substrates to confirm the activity of glycolysis. During those experiments, we have confirmed enzyme activity only in ETBF OMV but not in NTBF OMVs. Our finding and reconstructed maps indicate that ETBF OMVs could exist as an active, self-sufficient biological system. In this way, ATP and NADH—the main energy source in biochemical reactions—could be generated and fully used during ETBF vesicle persistence. Simultaneously, the generated ATP is not detected in the vesicle metabolome as the end product. We suggest that ATP and NADH could be utilized in vesicles during carbohydrate import by special ATP-dependent transporters or phosphorylation reactions such as phosphoribosyl pyrophosphate or fructose-1,6-bisphosphate synthesis or the TCA cycle. Several proteins that were detected in OMVs may utilize ATP during biochemical activity: mobility proteins, polynucleotide phosphorylase (PNPase) and histidine kinase^73, 74^. PNPase was found in *Salmonella enterica* as a factor contributing to bacterial invasion and intracellular replication by affecting the mRNA levels of a subset of virulence genes^75^. It should be noted that PNPase activity is modulated by the ATP in *E. coli*. We hypothesized that the ATP generated in ETBF OMVs regulates the activity of virulence factors, transport to other microorganisms, and cell-cell communication signals, as well as determines the distal pathogenicity of *B. fragilis*. Moreover in our previous study we have shown that ETBF toxin which is associated with OMVs causes epithelial cells contact disruption^46^. Thus the metabolic activity of the ETBF OMVs can facilitate its long-term contact with epithelial cells, increasing the damage level. However despite the possible role of ATP in mentioned processes it should be confirmed by future experiments.

In summary, we have characterized the vesicles of two genetically similar strains of *B. fragilis* and found a fundamental difference between them. NTBF OMVs have only enzymes needed for polysaccharide utilization, contributing to the nutrition function among bacterial types that lack this capability. By contrast, ETBF OMVs have active enzymes that possibly provide their stability and capability for long persistence, as well as contribute to virulence factors delivery. So we hypotized, that ETBF OMVs, during long persistence, can preserve and support enzyme activity by functioning as micro reactors and transmitting the information among other microbiota cells in the host. But this suggestion should be supported by further investigations in future.

## Material and Methods

### Bacterial strains and growth conditions

Enteropathogenic *Bacteroides fragilis* (ETBF) (*B. fragilis* strain BOB25)^44^ and non-toxigenic *B. fragilis* 323-J-86 (clinical isolates kindly provided from the Federal Research and Clinical Centre of Physical-Chemical Medicine Federal Medical Biological Agency, Moscow, Russia) were grown on blood agar plates containing either 5% defibrinated horse blood or brain heart infusion broth supplemented with hemin (5 g/ml) under anaerobic conditions.

### OMV purification

OMVs were isolated and purified from 24-h cultures of *Bacteroides fragilis* (BOB25 and JIM10) by multiple filtration and ultracentrifugation followed by visualization using transmission electron microscopy (TEM). Detailed information about the methods of OMV isolation, purification and visualization by TEM can be found in the Supplementary Information.

#### Cell fractionation

Cell fractionation was performed as described by Lindmark *et al*.^76^. Precipitated proteins from cell fractions were collected by centrifugation at 12,000 × *g*, washed with acetone, dried and dissolved in Laemmli sample buffer. The protein concentrations were quantified using a 2D-quant kit (GE Healthcare Life Sciences, USA).

### SDS PAGE and in-gel trypsin digestion of protein samples

Forty micrograms of each OMV sample (NTBF and ETBF) and 40–60 µg of cytoplasmic and membrane fractions were mixed with Laemmli sample buffer and were separated by SDS-PAGE. The resultant gels were cut into small (1 × 1 mm) pieces and were transferred into sample tubes. The samples were then subjected to in-gel trypsin digestion. Finally, the obtained peptides were dried in a vacuum and were dissolved in 3% ACN with 0.1% FA solution prior to HPLC-MS/MS analysis. Detailed information about the methods of SDS PAGE and in-gel trypsin digestion of protein samples can be found in the Supplementary Information.

### HPLC-MS/MS for proteome analysis

HPLC-MS/MS proteome analysis of OMVs was performed using a TripleTOF 5600+ mass spectrometer equipped with a NanoSpray III ion source (ABSciex, Canada) coupled to a NanoLC Ultra 2D+ nano-HPLC system (Eksigent, Dublin, CA). Comparative proteome analysis of vesicles and cells was performed using an Ultimate-3000 HPLC system (Thermo Scientific) coupled to a maXis qTOF after HDC-cell upgrade (Bruker) with a nanoelectrospray source. Detailed information about the parameters of HPLC-MS/MS analysis of tryptic peptides can be found in the Supplementary Information.

### Search Database Creation

The ProtDB search database was created using the annotated proteins of *Bacteroides fragilis* BOB25. Protein identification was carried out using the Mascot Search Engine version 2.5.1. Detailed information about Search Database Creation and protein and peptide identification can be found in the Supplementary Information.

### Proteogenomic Analysis

For proteogenomic analysis, the Genome Search Specific Peptides (GSSPs) and Protein database (ProtDB) software platforms were used. Reannotation of the coding sequences (CDSs) and open reading frames (ORFs) and pseudo gene search were carried out using the Prokka tool. Detailed information about proteogenomic analysis can be found in the Supplementary Information.

### Label-free protein quantification

For comparative analysis of the protein amount, Exponentially Modified Protein Abundance Indexes (emPAI) were calculated^77^. Data were normalized using the scaling method. Proteins were considered to be significantly different according to unpaired two-tailed Student’s t-test (p-value < 0.05) with Benjamini and Hochberg adjustment for p-values.

### Reagents for metabolome analysis

The following chemicals were used as standards for metabolome analysis: sodium pyruvate (100 mg/ml, disodium salt hydrate), D-fructose 6-phosphate, hydrate of sodium phospho(enol)pyruvate (97% purity, enzyme quality), dehydrated disodium salt of D-ribose 5-phosphate, and di-glyceraldehyde 3-phosphate (46.1 mg/ml). Purified (98%) amino acids, nucleotides, nucleosides (adenosine, deoxyadenosine, inosine, cytosine monophosphate, and thymidine) from Sigma-Aldrich (USA) were also used as standards. The following reagents were used for extraction and solution preparation: absolute methanol (HPLC grade) from Biosolve (The Netherlands), ammonium acetate (ultra clean grade) from Helicon (Russia), formic acid (98–100%) from Riedel-de Haen (Germany), ammonium hydroxide solution (29.73%) from Fisher Scientific (USA), water (HPLC-MS) and acetonitrile (HPLC-MS) from Panreac (Spain).

### LC-MS analysis and metabolite identification

Metabolite extraction was carried out using the cold methanol method with several modifications (described in the Supplementary Information)^78–80^. Metabolite analysis was performed using an LC-MS-8030 triple quadrupole liquid chromatography system (Shimadzu, Kyoto, Japan). The instrument control and data processing were performed using the workstation “LabSolutions LCMS” Version 5.75 (Shimadzu Corporation, Kyoto, Japan). The metabolites were analyzed in the multiple reaction monitoring (MRM) modes, with two transitions per compound for identification and quantification purposes. The identification of qualitative differences in metabolites isolated from the OMVs of ETBF and NTBF was performed using paired multiple-adjusted t-test. To account for multiple testing and to control the false discovery rate (FDR), the Benjamini–Hochberg procedure was used^81^. A cut-off value for FDR (*q* < 0.2) was applied according to previous metabolomic studies^82–84^. Detailed information about LC-MS parameters and metabolite identification can be found in the Supplementary Information.

### GC-MS method for metabolite analysis and identification

For the GS-MS method, the procedure for OMV metabolite extraction was the same as that described for LC-MS analysis in the Supplementary Information with several modifications. The resultant freeze-dried samples were dissolved in 20 µl of pyridine and were converted to trimethylsilyl derivatives. Gas chromatography-mass spectrometry and GC-MS data analysis of silylated samples were analyzed using an Agilent 7890 gas chromatograph interfaced with an mass selective detector 5975 C (MSD). The data were processed with UniCrom (New Analytical Systems (Belarussia)) and quantified with the AMDIS (http://chemdata.nist.gov/mass-spc/amdis/downloads/) and NIST8, Wiley9 and GOLM (http://gmd.mpimp-golm.mpg.de) software’s.

### Fluxome experiments

Solutions containing freshly isolated ETBF and NTBF OMVs were separated into three equal parts. One microgram of D-Glucose ^13^C was added to previously prepared OMV-containing solutions and was incubated at 37 °C for 30 min, 1 and 3 h. One microgram each of glucose 1, 3 (C13) and glucose 6 (C13) was also added to OMV-free 150 mM NaCl solution as the negative control. After incubation, all of the OMV preparations were subjected to ultracentrifugation at 100,000 *g* for 2 h (Optima L-90K ultracentrifuge; Beckman Coulter). The supernatant was discarded, and then the pellet was dissolved in 150 mM NaCl and prepared for metabolome extraction as described earlier in the “**Extraction of metabolites**” section.

## Electronic supplementary material

Supplementary information

Supplementary table S1

Supplementary table S2

Supplementary table S3-1

Supplementary table S3-2

Supplementary table S3-3

Supplementary table S3-4

Supplementary table S3-5

Supplementary table S3-6

Supplementary table S3-7

Supplementary table S4

Supplementary table S5

Supplementary table S6
