# Supplementary material for: Outer membrane vesicles secreted by pathogenic and nonpathogenic Bacteroides fragilis represent different metabolic activities
Source: Sci Rep. 2017 Jul 10;7:5008. doi: 10.1038/s41598-017-05264-6 (PMC5503946; doi:10.1038/s41598-017-05264-6)
Supplement: Supplementary file 1 — Supplementary information [file 41598_2017_5264_MOESM1_ESM.doc]

**Outer membrane vesicles secreted by pathogenic and nonpathogenic *Bacteroides fragilis* represent different metabolic activities**

Natalya B. Zakharzhevskaya^1#^, Anna A. Vanyushkina^1^, Ilya A. Altukhov^2^, Aleksey L. Shavarda^3,4^, Ivan O. Butenko^1^, Daria V. Rakitina^1^, [Anastasia S. Nikitina](http://www.ncbi.nlm.nih.gov/pubmed/?term=Nikitina AS%5Bauth%5D)^1^, [Aleksandr I. Manolov](http://www.ncbi.nlm.nih.gov/pubmed/?term=Manolov AI%5Bauth%5D)^1^, Alina N. Egorova^1,2^, Eugene E. Kulikov^2,8^, A. [Innokentii E. Vishnyakov](http://www.frontiersin.org/people/u/394683)^6,7^, Gleb Y. Fisunov^1^, Vadim M. Govorun^1,2,5^

- 1-Federal Research and Clinical Centre of Physical-Chemical Medicine Federal Medical Biological Agency, Malaya Pirogovskaya str., 1a, Moscow 119435, Russian Federation; 2-Moscow Institute of Physics and Technology, Institutskiy Pereulok 9, Dolgoprudny 141700, Russian Federation; 3 – Research Resource Center Molecular and Cell Technologies, Saint-Petersburg State University, Universitetskaya nab. 7-9, Saint-Petersburg 199034, Russian Federation; 4 - Analytical Phytochemistry Laboratory, Komarov Botanical Institute, Prof. Popov Street 2, Saint-Petersburg 197376, Russia; 5 - Shemyakin-Ovchinnikov Institute of Bioorganic Chemistry, Miklukho-Maklaya str. 16/10, Moscow 117997, Russian Federation, Moscow, Russia; 6- Lab of Genome Structural Organization, Institute of Cytology, Russian Academy of Sciences, Saint Petersburg, Russia;7- Institute of Nanobiotechnologies, Peter the Great St. Petersburg Polytechnic University, Saint Petersburg, Russia;8- Microbial viruses laboratory, Research Center of Biotechnology RAS;

***Genomes alignment***

To find differences between genomes (BOB25 and JIM10) of the two strains we mapped reads in reciprocal way with bowtie2. SNPs were evaluated using samtools mpileup and varscan with p-value threshold equals 10^-5^, minimum four reads coverage and frequency threshold equals 0.9. Then custom R script was used to calculate SNP density in a sliding window of 10000 nucleotides. Modal value was used as estimation of SNP rate. To find unique genes reads count per gene were calculated using bedtools and reads with zero reads mapping to them were assumed as unique.

***OMV isolation and purification***

Two hundred fifty milliliters of 24-h cultures of *Bacteroides fragilis* (ETBF and NTBF) were centrifuged at 4,500*g* at 4°C. In order to remove residual cells, the supernatant was filtered using a 0.45 µm pore membrane. (Millex GV; Millipore) The filtrate was subjected to ultracentrifugation at 100,000 *g_n_* for 2 h (Optima L-90K ultracentrifuge; Beckman Coulter). The supernatant was discarded; the pellet was washed with sterile PBS and filtered through a sterile 0.2 µm-pore polyvinylidene difluoride (PVDF) membrane (Millex GV; Millipore). The ultracentrifugation step was repeated twice.

The vesicle pellet was resuspended in distilled water or 150 mM NaCl (pH 6.5). Protein concentration was quantified using the 2D-quant kit (GE Healthcare Life Sciences).

***OMV purification using sucrose gradient***

Two hundred fifty milliliters of 24-h cultures of *Bacteroides fragilis* (ETBF and NTBF) were centrifuged at 4,500*g* at 4°C. In order to remove residual cells, the supernatant was filtered using a 0.45 µm pore membrane (Millex GV; Millipore). The filtrate was subjected to ultracentrifugation at 100,000 *g_n_* for 2 h (Optima L-90K ultracentrifuge; Beckman Coulter). The supernatant was discarded; the pellet was washed with sterile PBS and subjected to sucrose step gradient sedimentation (30%-50%). Collected opalescent layer was subjected to ultracentrifugation at 100,000 *g_n_* for 2 h (Optima L-90K ultracentrifuge; Beckman Coulter). The ultracentrifugation step was repeated twice. The vesicle pellet was resuspended in distilled water or 150 mM NaCl (pH 6.5). Protein concentration was quantified using the 2D-quant kit (GE Healthcare Life Sciences).

***Electron microscopy***

Ultrathin sections of ETBF and NTBF were prepared as previously described ^77^. 5 µl of each samples were negatively stained with 2% (wt/vol) uranyl acetate for 3 min and examined using a Zeiss Libra 120 electron microscope (Zeiss, Germany). OMV sizes were determined using the images obtained

***SDS PAGE and In-gel trypsin digestion of protein samples***

The isolated OMVs and cells from ETBF and NTBF were mixed with Laemmli sample buffer (1:1) containing CHAPS and separated by SDS-PAGE. 40 µg of each (NTBF and ETBF) OMV samples and 40-60 µg of each cells lysate from ETBF and NTBF were boiled for 10 min prior to electrophoresis. Gels were stained with Coomassie brilliant blue stain. Gel were cut into small (1 × 1 mm) pieces and transferred into sample tubes. Protein disulfide bonds were reduced with 10 mM DTT (in 100 mM ammonium bicarbonate buffer) at 50^○^ C for 30 min and afterwards alkylated with 55 mM iodoacetamide (in 100 mM ammonium bicarbonate buffer) at room temperature for 20 min in the dark. After alkylation, gel samples were stained with 50% ACN (in 50 mM ammonium bicarbonate buffer) and dehydrated by addition of 100% ACN. After removal 100% ACN, the samples were subjected to the in-gel trypsin digestion. The digestion buffer contained 13 ng/µl trypsin (in 50 mM ammonium bicarbonate buffer). The trypsin digestion proceeded overnight at 37^○^ C. The resulting tryptic peptides were extracted from the gel by adding two volumes of 0.5% TFA into the samples (incubation during1 h), and, then, two volumes of 50% ACN (incubation during 1 h). Finally, the extracted peptides were dried in vacuum and redissolved in 3% ACN with 0.1% FA solution prior to LC-MS/MS analysis.

***HPLC-MS/MS parameters for OMVs proteome analysis***

The HPLC system was configured in a trap-elute mode. For sample loading Buffer A (98.9% water, 1% methanol, 0.1% formic acid(v/v)) was used. Elution Buffer B was 99.9% acetonitrile, 0.1% formic acid (v/v). Samples were loaded on a trap column Chrom XP C18 (120 Å) 3 µm, 0.5 mm * 350 µm at a flow rate of 3 µl/min for 10 min and eluted through the separation column 3C18-CL-120 (120 Å) 3 µm, 150 mm * 75 µm (Eksigent, Dublin, CA) at a flow rate of 300 nl/min. The gradient was from 5 to 40% of buffer B in 120 min or from 5% (0 min) to 27.3% (75 min) to 40% (85 min) of buffer B while the first part of this gradient – from 5% to 27.3% of buffer B – had the same slope as a 5-to-40% B gradient. The analytical column and guard column were regenerated between runs by washing with 95% buffer B for 7 min and equilibrated with 5% buffer B for 25 min. To ensure the absence of cross-contamination between samples, both the column and the guard column were thoroughly washed with a trap-elute gradient that included five cycles of the following steps: 5% (0 min time point) to 95% (3 min time point) to 95% (6 min time point) to 5% (7 min time point) to 5% (10 min time point – the 0 min time point of the next cycle) of buffer B.

The information-dependent mass spectrometer experiment included 1 survey MS1 scan followed by 50 dependent MS2 scans. MS1 acquisition parameters were as follows: mass range for analysis and subsequent ion selection for MS2 analysis was 300-1250 m/z, signal accumulation time was 250 ms. Ions for MS2 analysis were selected on the basis of intensity with the threshold of 250 cps and the charge state from 2 to 5. MS2 acquisition parameters were as follows: resolution of quadrupole was set to UNIT (0.7 Da), measurement mass range was 200-1800 m/z, optimization of ion beam focus was adjusted to obtain maximal sensitivity, signal accumulation time was 50 ms for each parent ion. Collision activated dissociation was performed with nitrogen gas with collision energy ramping from 25 to 55 V within 50 ms signal accumulation time. Analysed parent ions were set to dynamic exclusion list for 15 sec to obtain a MS2 spectra at the chromatographic peak apex (minimum peak width throughout the gradient was approximately 30 s).

***Comparative LC-MS/MS analysis of cells and OMVs proteome***

LC-MS/MS analysis of tryptic peptides was carried out using Ultimate-3000 HPLC system (Thermo Scientific) coupled to a maXis qTOF after HDC-cell upgrade (Bruker) with a nano-electrospray source. Chromatographic separation of peptides was performed on a C-18 reversed phase column (Zorbax 300SB-C18, 150 mm x 75 um, particle diameter 3.5 um, Agilent). Gradient parameters were as follows: 5-35% acetonitrile in aqueous 0.1% (v/v) formic acid, duration 120 min, column flow 0.3 ul/min. Positive MS and MS/MS spectra were aquired using AutoMS/MS mode (capillary voltage 1700, curtain gas flow 4 l/min, curtain gas temperature 170 C, spectra rate 10 Hz, 4 precursors, m/z range 50-2200, active exclusion after 2 spectra, release after 0.5 min).

***Search Database Creation***

Annotated proteins were downloaded in fasta format (RefSeq: NZ_CP011073.1, 4127 amino acid sequences). The genome was downloaded from NCBI in fasta format (NZ_CP011073.1) and was translated in 6-frames. Stop-to-stop ORFs were exported using the Artemis software version 16.0.0 with option “Mark Open Reading Frames” ^78^. Minimal ORF length was set at 40 amino acids.

***Proteins and Peptides Identification***

Raw data files with WIFF and .D file format were converted to the Mascot generic format (MGF file format) using AB SCIEX MS Data Converter version 1.3 and Compass Data Analysis 4.2 (Build 383.1) respectively. The proteins identification was carried out using Mascot Search Engine version 2.5.1.The Mascot searches were performed with the following parameters: tryptic-specific peptides, maximum of one missed cleavages, a peptide charge state limited to 1+, 2+ and 3+ a peptide mass tolerance of 10 ppm, a fragment mass tolerance of 0.5 Da, variable modifications caused by Oxidation(M) and Propionamide(C). The decoy search strategy to calculate FDR was used. The score threshold was calculated using Mascot. Individual ions score higher than score threshold indicate identity or extensive homology with p < 0.05 and FDR < 5%. A peptide was identified if its rank was 1 and score higher than score threshold. A protein was identified if has 2 and more identified unique peptides. The mass spectrometry proteomics data have been deposited to the ProteomeXchange Consortium (Project accession: PXD005255) via the PRIDE partner repository with the dataset identifier PXD.

***Proteogenomic Analysis***

MS/MS data were searched against a six-frame translated genome sequence (GenDB) to identify novel protein-coding regions. After excluding peptides that identified by protein database (ProtDB), the GSSPs (Genome Search Specific Peptides) were further analyzed to refine current genome annotation ^79^. GSSPs were categorized in three groups: (1) peptides mapping to ORF, which does not contain a gene, (2) peptides mapping to ORF, which contains a gene, (3) peptides mapping to ORF, which contains pseudogene.

ORF, which contains at least 2 GSSPs and does not include gene, was marked as CDS-containing regions. Start-codon of CDS, which contains at least one GSSP in the same ORF, was reannotated. CDS reannotation was carried out using Prokka tool with default parameters ^80^.

Sequencing errors were searched in regions, which contain GSSP and pseudogene in the same strand. ORFs that are in the pseudogene environment (±1000 bp) and have the same strand were aligned to NCBInr database using BLASTP algorithm. ORFs aligned on the same protein were selected for analysis on sequencing errors and frame shifts.

***Metabolites extraction***

For metabolite analysis OMVs were extracted from 250 ml ETBF and NTBF cell culture grown in a liquid medium to logarithmic phase. OMVs were washed in 150 mM NaCl and harvested by ultracentrifugation at 100,000 g at 4°C for 1 hour. The OMVs pellet was resuspended in 5 ml of 150 mM NaCl and precipitated again by ultracentrifugation at 100,000 g at 4°C for 1 hour. Resulted OMVs pellet was resuspended in 100 µl ml of 150 mM NaCl.

The OMVs metabolic action was rapidly quenched by cold methanol. A cold methanol extraction method was developed on the basis of a previously reported cold methanol extraction protocol as described below. Briefly the metabolites were extracted by adding 900 µl of methanol (−20°C) to 100 µl of the resuspended OMVs in 150 mM NaCl; 10 µl of isotope labeled 1 mg/ml L-Glutamic acid-13C5 (Sigma-Aldrich, Saint-Louis, USA) was added to mix as internal standard; sample was vigorously shaken (1 min) and then kept at −77°C for 15 min. The sample was thawed during 3 min at room temperature and then thoroughly shaken again. The resulting sample was centrifuged for 30 min at 16,000 g at 4°C. The supernatant was vacuum dried using SpeedVac concentrator (Thermo Fisher Scientific™, USA). The dry extract was kept no longer than 10 days prior to analysis. The dry extract was dissolved in 100 µl of mixture consisting of 20% acetonitrile and 80% water and analyzed immediately.

### *HPLC/MS method for metabolites analysis*

### Metabolite analysis was performed on a LCMS-8030 triple quadrupole liquid chromatography system (Shimadzu, Kyoto, Japan). The eluent flow from the analytical column was introduced directly into the electrospray ion source of the mass spectrometer. The ionizing spray voltage was 4500 V in both positive and negative ionization modes. Nitrogen of various degrees of purity was used as a dry gas flow rate of 3 l/min, as a nebulizing gas with flow rate 13 l/min and as a CID gas with a pressure 17 kPa. The temperature of the desolvation-line unit was 250°C, the heat block temperature was 400°C.

Mass spectrometry metabolite analysis was performed in MRM (multiple reaction monitoring) mode with following parameters in positive ionization mode: 53 MRM events, dwell time of each transition acquisition was 10 ms, maximum loop time was 2.245 s, 5 time segments were used as showed in table 1. And in negative ionization mode: 61 MRM events, dwell time of each transition acquisition was 10 ms, maximum loop time was 2.455 s, 11 time segments were used as showed in table 1. Resolution of Q1 and Q3 were unit for both acquisition modes. Time segments of MRM analysis, collision energy and retention time of different metabolites was selected based on HPLC-MS/MS analysis of chemical standards. Metabolite separation was carried out using the high performance liquid chromatography (Shimadzu, Kyoto, Japan). The following chromatographic analytical column was used in the study: Zorbax RX-SIL Narrow-Bore (150 mm×2.1 mm×5 µm) equipped with guard column Zorbax RX-SIL 4-Pack (4.6 mm×12.5 mm×5 µm) from Agilent Technologies.

Chromatographic analysis was performed with the following parameters: auto-sampling temperature, 20°C; analytical column temperature, 32°C; injection volume, 10 µl; solvent flow rate, 500 µl/min. The following solvents were used as eluting solutions: eluent A was 20 mM ammonium acetate/0.25 mM ammonium hydroxide in water/acetonitrile mixture of 95∶5 ratio, pH 8.00; eluent B was pure acetonitrile. The gradient of the solvent transition was as follows: for positive and negative ionization mode t = 0 min, 100% B; t = 15 min, 0% B; t = 18 min, 0% B; t = 19 min, 100% B; t = 32 min, 100% B.

### *Identification of metabolites and data processing software*

The instrument control and the data processing were done by workstation “LabSolutions LCMS” Version 5.75 (Shimadzu Corporation, Kyoto, Japan). Metabolites were analyzed in the multiple reaction monitoring (MRM) mode, with two transitions per compound for identification and quantification purposes. [M+H]+ and [M-H]- served as precursor ion, and the most intense transition was used to the quantify difference in sample extracts. Identification of metabolites was based on the following criteria: the retention time of the analyte ± 0.5 min; the presence of two fragments. We used following peak integration parameters: Width - 30 sec, Slope -1000/min, Min Area/Height 10 counts, Noise/Drift detection limit - 3 and Quantification limit – 10. To compare metabolites content of different samples was performed signal normalization using total protein content value. For MS signal normalization the relative coefficient was determined with isotopically labeled internal standard. L-Glutamate- ^13^C_5_ solution as described earlier. Identification of qualitative differences in toxigenic and non-toxigenic strains performed using paired multiple-adjusted t-test. The metabolic quantity difference between two groups (ETBF and NTBF) was examined using paired Student's t-test at the *p* value level of 0.05. To account for multiple testing and for control false discovery rate (FDR) the Benjamini–Hochberg procedure was used ^73^. A cut-off value for FDR (q < 0.2) was applied according to previous metabolomic studies ^74-76^. The mass spectrometry metabolomic data have been deposited to UCSD Center for Computational Mass Spectrometry (MassIVE ID: MSV000080304).

### *GC-MS method for metabolites analysis and identification*

For metabolite analysis OMVs were extracted from 250 ml ETBF and NTBF cell culture grown in a liquid medium to logarithmic phase. OMVs were washed in 150 mM NaCl and harvested by ultracentrifugation at 100,000 g at 4°C for 1 hour. The OMVs pellet was resuspended in 5 ml of 150 mM NaCl and precipitated again by ultracentrifugation at 100,000 g at 4°C for 1 hour. Resulted OMVs pellet was resuspended in 100 µl ml of 150 mM NaCl. Metabolites were extracted by adding 900 µl of methanol (−20°C) to 100 µl of the resuspended OMVs in 150 mM NaCl; 10 µl of isotope labeled 1 mg/ml L-Glutamic acid-13C5 (Sigma-Aldrich, Saint-Louis, USA) was added to mix as internal standard; sample was vigorously shaken (1 min) and then kept at −77°C for 15 min. The sample was thawed during 3 min at room temperature and then thoroughly shaken again. The resulting sample was centrifuged for 30 min at 16,000 g at 4°C. The supernatant was vacuum dried using SpeedVac concentrator (Thermo Fisher Scientific™, USA).

Resulted freeze-dried samples were dissolved in 20 µl of pyridine and converted to trimethylsilyl derivatives by adding 20 µl of N,O-bis-(trimethylsilyl) trifluoroacetamide (BSTFA) containing 1% trimethylchlorosilane (TMCS). The chemical reaction was performed by heating to 100^○^ C for 15 min, 0.5 µl of this reaction mixture was injected into the gas chromatograph. Gas chromatography – mass spectrometry and GC-MS data analysis silylated samples were analyzed using an HP 6850 gas chromatograph interfaced with an HP 5975C mass selective detector (MSD). An HB5-MS capillary column (30 m x 0.25 mm I. D. and film thickness of 0.25 µm) was used with helium as a carrier gas at a constant rate of 1 ml/min. The temperatures of the injector and MS source were maintained at 320 and 230^○^ C, respectively. The column temperature program consisted of injection at 70○ C with an increase of 6^○^ C/min up to 320^○^ C followed by an isothermal hold at 320^○^ C for 15 min. Tricosane (10 µg) was used as an internal standard for quantification of analytical results. The samples were analyzed in split mode (split ratio: 1/20). The MS was operated in the electron impact mode with ionization energy of 70 eV. The scan mass range was set from 50 to 1000 Da at 1.27 scan/sec. The data were processed and quantified with the AMDIS software (http://chemdata.nist.gov/mass-spc/amdis/downloads/). Compound identification was performed by comparison with the chromatographic retention characteristics and mass-spectra of authentic standards, reported mass spectra, and the mass spectral library of the GC–MS data system (NIST 8 2010). The sum of Extracted Ion Chromatograms (XIC) of the ions associated with a compound was used for quantification.

**SUPPLEMENTARY FIGURE LEGENDS**

**
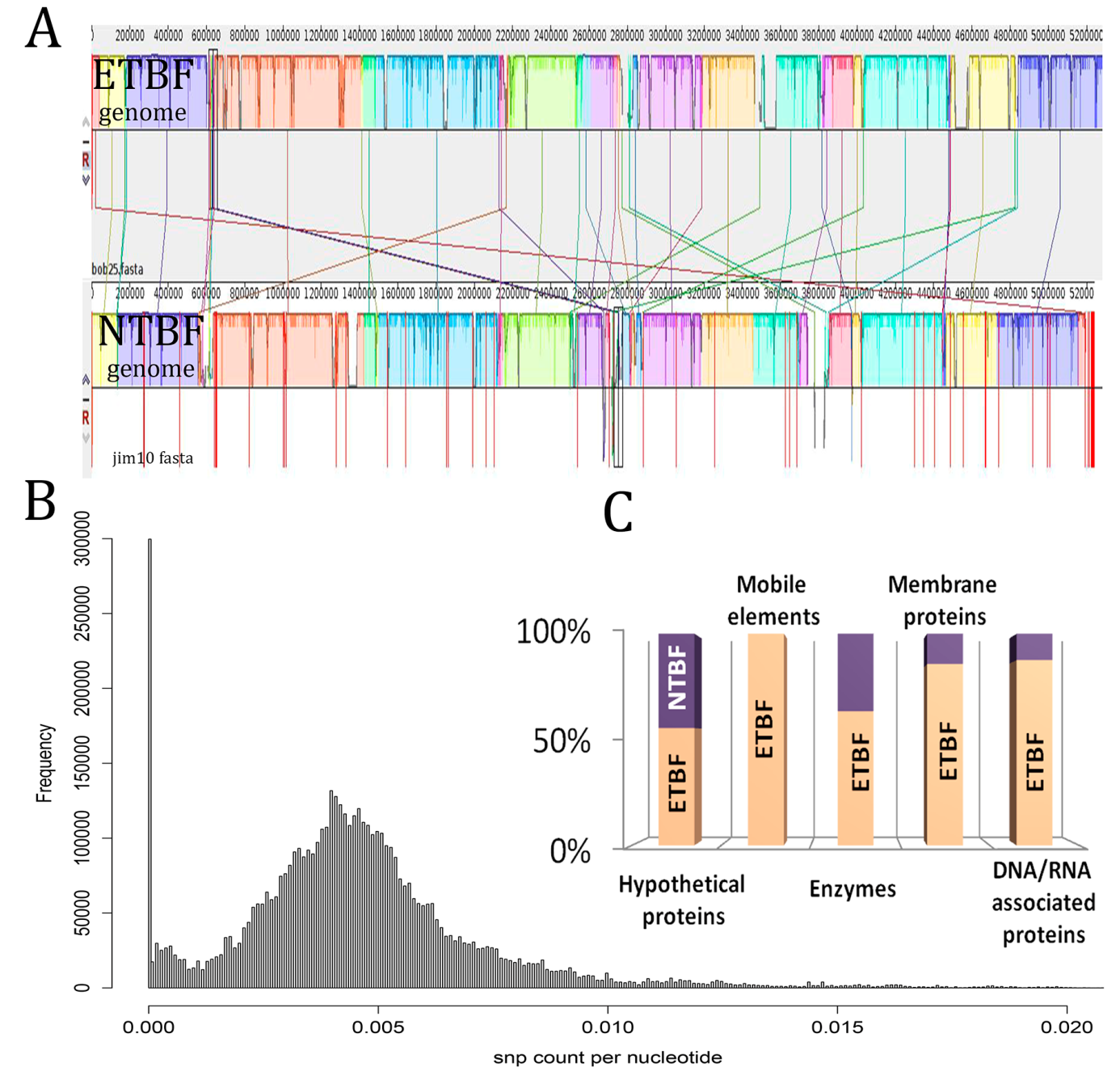
**

Supplementary figure S1 Alignment of two genomes (BOB25 and JIM10) prepared using Mauve program. (A) – comparative analysis of BOB25 and JIM10 genomes. (B) - SNP rate provided for BOB25 and JIM10 describes the genome difference between two strains (C) – The main genomes differences between two strains in percentage relative to each other.


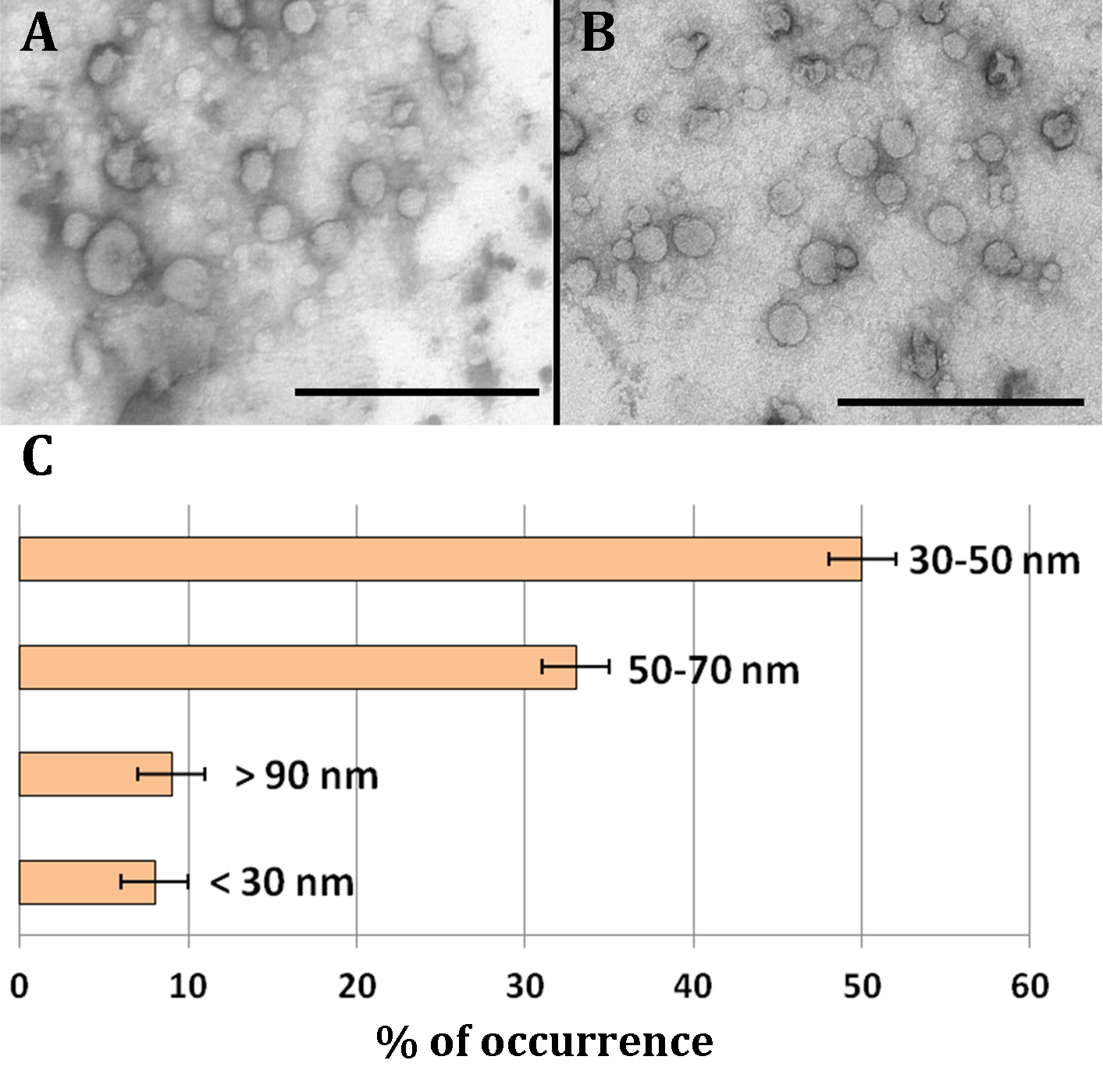


**Supplementary figure S2 ETBF and NTBF produce outer membrane vesicles (OMV)** **(A-B)** TEM of negatively stained OMVs isolated from cell free culture medium (**A – ETBF and B - NTBF**), at magnification: x 10,000. Scale bars represent 1µm for **(A,B)**, 300 nm. **(C)** - OMV size distribution diagram determined from measurements of about 1000 OMVs from 10 samples


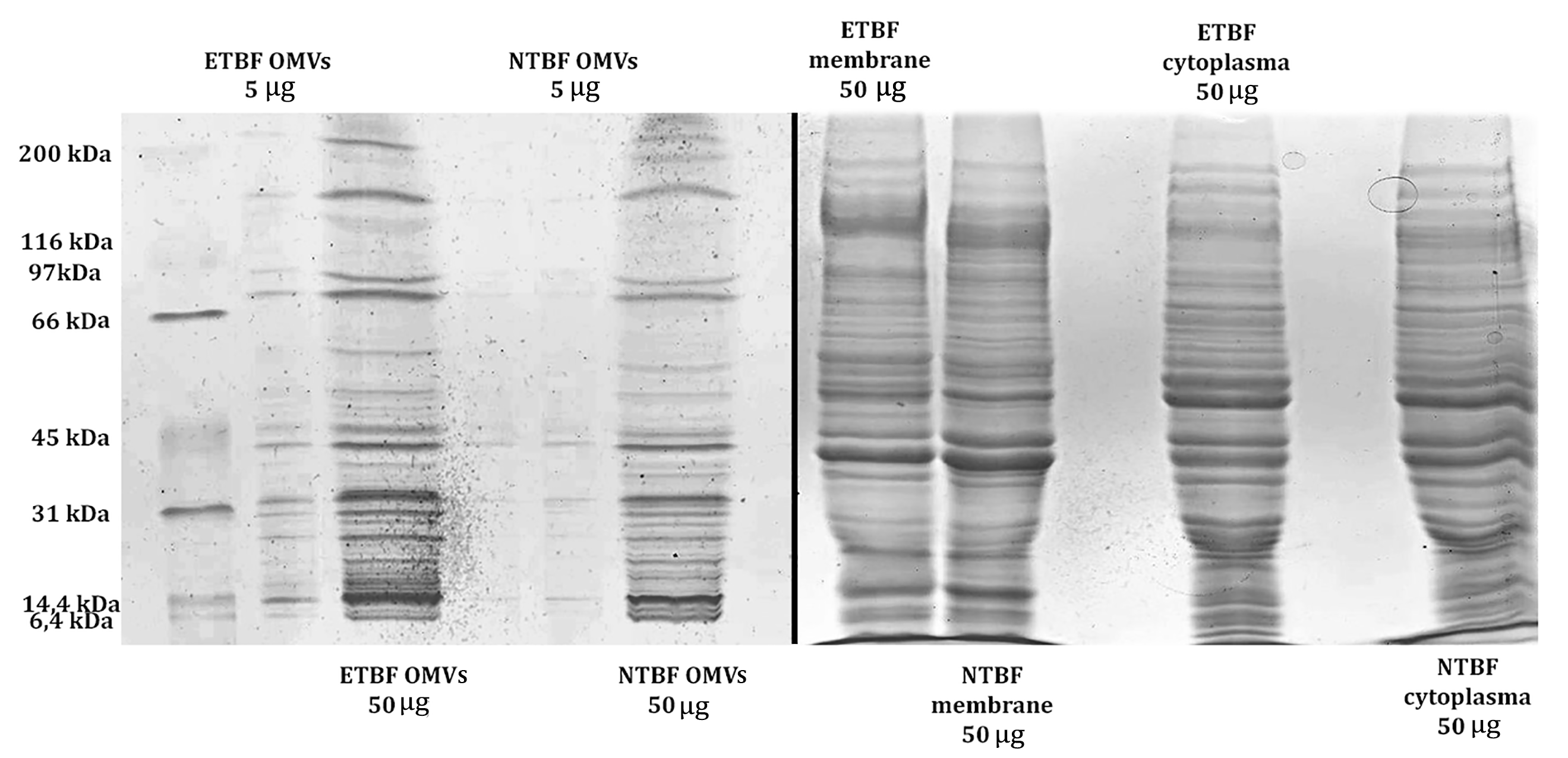


**Supplementary figure S3 ETBF and NTBF OMV**s show different protein profile from that of ETBF and NTBF cytoplasm and membrane fraction. 5 and 50 micrograms of purified ETBF and NTBF OMV and 5 and 50 micrograms of extracted ETBF and NTBF cytoplasm and membrane fraction were run on 10% SDS-PAGE followed by Coomassie staining. Protein bands were excised from the gel and digested with trypsin. The resulting peptides were enriched using ZipTip C18 columns then analyzed via liquid chromatography coupled to tandem mass spectrometry (HPLC-MS/MS) followed by protein identification with Mascot search engine using the NCBInr database.


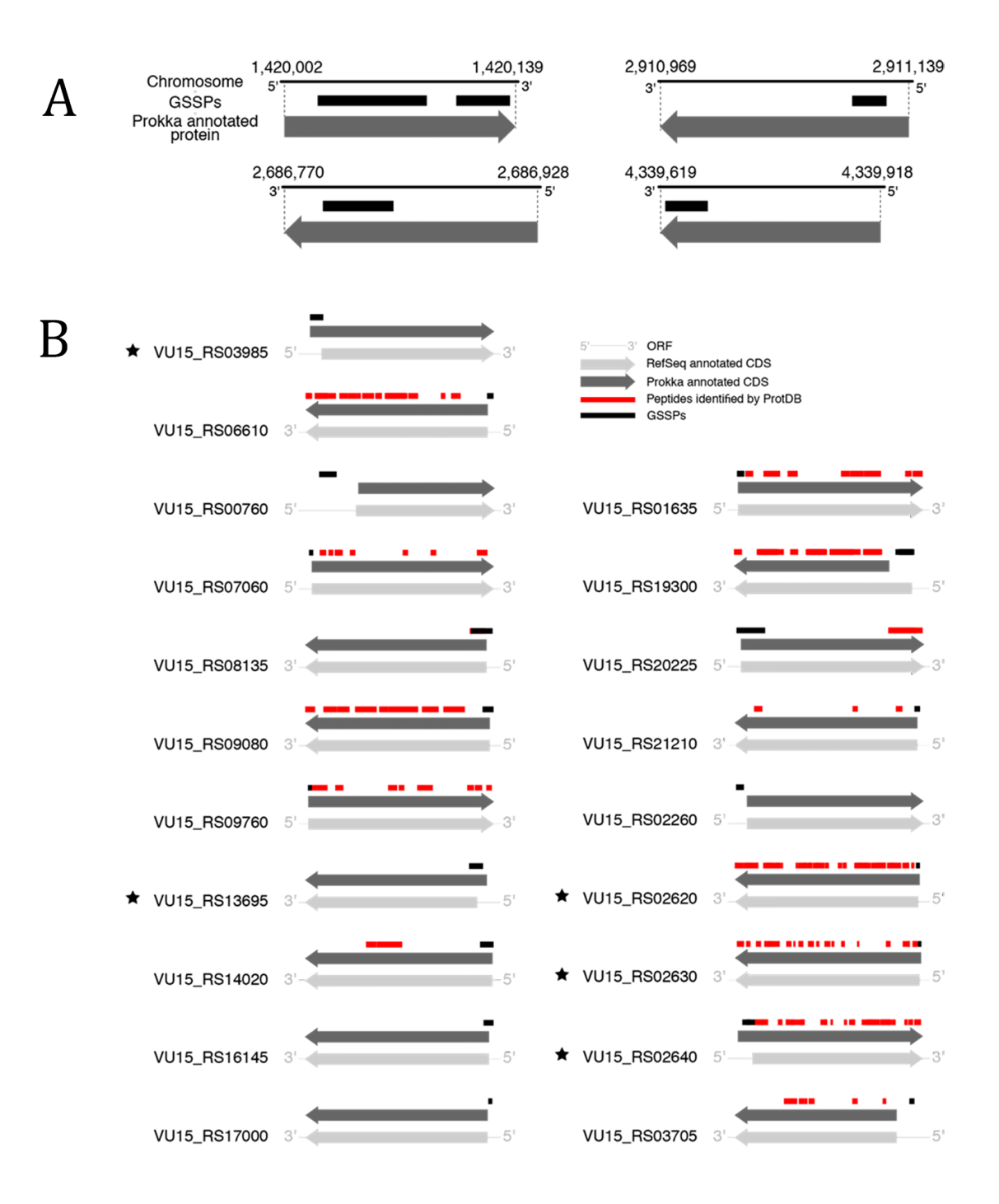


**Supplementary figure S4 Identification of Novel Protein Coding Genes Using Genome Search Specific Peptides (A) -** The novel identified protein-coding genes in *B.fragilis* BOB25. **(B)** - 27 GSSPs are identified in 20 CDS-containing ORFs. Genes are predicted using Prokka tool. 5 CDSs from Prokka annotation included GSSPs


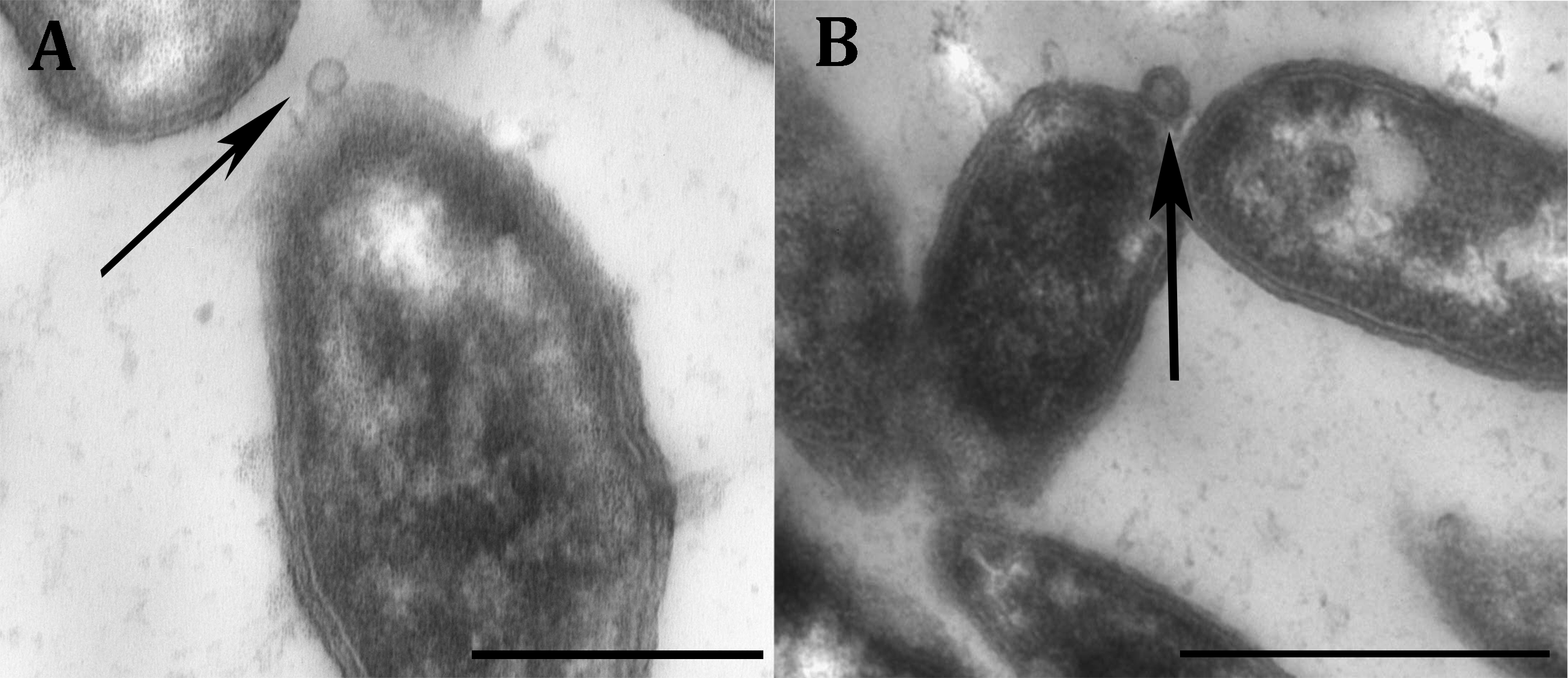


**Supplementary figure S5 ETBF produce different types of vesicles (OMV)** **(A)** TEM of negatively stained cell produced single membrane OMV, at magnification: x 10,000. Scale bars represent 500 nm for **(A)**; **(B**) TEM of negatively stained cell produced double membrane OMV, at magnification: x 10,000. Scale bars represent 1µm for **(B)**


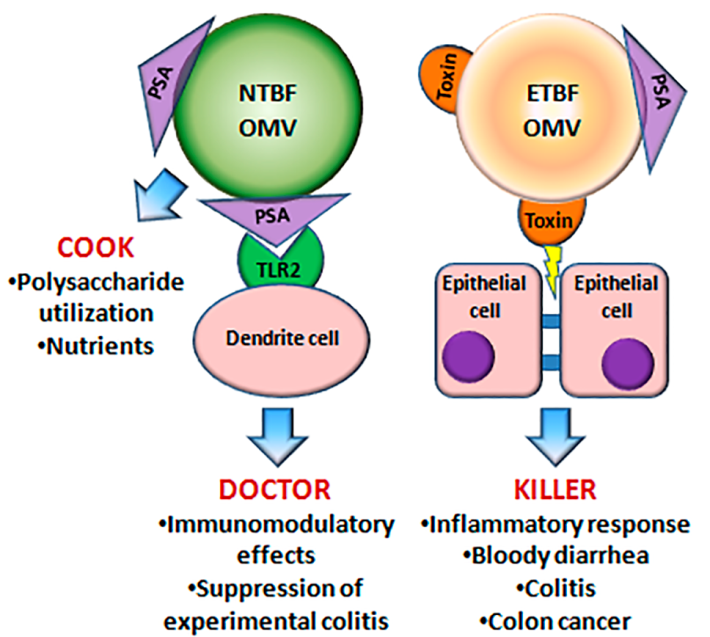


**Supplementary figure S6 Schematic sketch of the main effects of the vesicles produced by ETBF and NTBF** Figure represents the main described effects of *B.fragilis* OMVs (High hydrolase activity against surface located polysaccharides) and newly described properties (Virulence factors delivery)
